# ABC-transporter CFTR folds with high fidelity through a modular, stepwise pathway

**DOI:** 10.1007/s00018-022-04671-x

**Published:** 2023-01-07

**Authors:** Jisu Im, Tamara Hillenaar, Hui Ying Yeoh, Priyanka Sahasrabudhe, Marjolein Mijnders, Marcel van Willigen, Azib Hagos, Eduardo de Mattos, Peter van der Sluijs, Ineke Braakman

**Affiliations:** 1 Cellular Protein Chemistry, Faculty of Science, Bijvoet Centre for Biomolecular Research, Science for Life, Utrecht University, Padualaan 8, 3584 CH Utrecht, The Netherlands; 2 Present Address: Center of Translational Immunology, University Medical Center Utrecht, Utrecht, The Netherlands; 3 Present Address: Navigo Proteins GmbH, 06120 Halle, Germany; 4 Present Address: Princess Máxima Center for Pediatric Oncology, 3584 CS Utrecht, The Netherlands; 5 Present Address: Julius Clinical Ltd, 3703 CD Zeist, The Netherlands

**Keywords:** Protein folding, Domain assembly, COPII, Secretory pathway, ABC-transporter, Cystic fibrosis

## Abstract

The question how proteins fold is especially pointed for large multi-domain, multi-spanning membrane proteins with complex topologies. We have uncovered the sequence of events that encompass proper folding of the ABC transporter CFTR in live cells by combining kinetic radiolabeling with protease-susceptibility assays. We found that CFTR folds in two clearly distinct stages. The first, co-translational, stage involves folding of the 2 transmembrane domains TMD1 and TMD2, plus one nucleotide-binding domain, NBD1. The second stage is a simultaneous, post-translational increase in protease resistance for both TMDs and NBD2, caused by assembly of these domains onto NBD1. Our assays probe every 2–3 residues (on average) in CFTR. This in-depth analysis at amino-acid level allows detailed analysis of domain folding and importantly also the next level: assembly of the domains into native, folded CFTR. Defects and changes brought about by medicines, chaperones, or mutations also are amenable to analysis. We here show that the well-known disease-causing mutation F508del, which established cystic fibrosis as protein-folding disease, caused co-translational misfolding of NBD1 but not TMD1 nor TMD2 in stage 1, leading to absence of stage-2 folding. Corrector drugs rescued stage 2 without rescuing NBD1. Likewise, the DxD motif in NBD1 that was identified to be required for export of CFTR from the ER we found to be required already *upstream* of export as CFTR mutated in this motif phenocopies F508del CFTR. The highly modular and stepwise folding process of such a large, complex protein explains the relatively high fidelity and correctability of its folding.

**Supplementary Information:**

The online version contains supplementary material available at 10.1007/s00018-022-04671-x.

## Introduction

Membrane proteins account for one-third of all of the proteins encoded by the genome of sequenced species. They mediate and integrate fundamental processes occurring on both sides of biological membranes. In humans at least 40% of membrane proteins span the membrane more than once [1]. These so-called polytopic membrane proteins include receptors, transporters, and channels amongst others and are the target of more than half of all small-molecule drugs [1, 2]. Many inherited diseases are associated with impaired folding and function of membrane proteins, signifying the importance of uncovering their underlying folding mechanisms for human health and drug development [3].

Folding of polytopic membrane proteins in vivo can be viewed as a series of sequential overlapping steps [4]. While the nascent chain is translated by ER-associated ribosomes, transmembrane helices (TMHs) are inserted and integrated into the ER membrane. Helical packing within the membrane occurs, along with folding of soluble domains and their organization into a functional protein. Co-translational folding intermediates have been characterized for ribosome-bound nascent chains [5, 6] and domains in a few multi-domain membrane proteins and have been found to fold individually and co-translationally [5, 7, 8]. Yet, how de-novo synthesized transmembrane domains fold and assemble into a mature, functional structure is still largely unknown.

We have addressed this question using the ABC transporter CFTR (cystic fibrosis transmembrane conductance regulator) as a model protein. The ABC transporter superfamily is one of the oldest and most conserved protein superfamilies [9, 10]. ABC transporters are multi-domain, multi-spanning membrane proteins, which transport various substrates across membranes, regulated by ATP hydrolysis, and are, therefore, crucial for homeostasis. CFTR functions as a chloride channel in the plasma membrane [11], and mutations in CFTR cause the disease cystic fibrosis (CF) [12].

CFTR contains two similar halves with each a transmembrane domain (TMD) and a nucleotide-binding domain (NBD). The halves are connected via the intrinsically unstructured Regulatory (R) region. The cryo-EM structures and various structural models of CFTR show that the TMDs are assembled via their transmembrane helices and helical intracellular loops (ICLs) [13–17]. The end of the ICLs, the so-called coupling helices, connect the TMDs with the NBDs. Each TMD contains two ICLs, one of which interacts with the NBD in the same half of the molecule whereas the other ICL binds to the NBD in the other half, in a cross-over fashion.

Seminal studies uncovered the orientation and integration of CFTR transmembrane helices into the membrane [18–20]. The signal recognition particle recognizes signal(s) in CFTR transmembrane helices and targets them to the translocon, for insertion into the ER membrane. The polypeptide sequence around transmembrane helices of polytopic proteins can cause translocation stop or re-start to allow formation of cytosolic and lumenal domains during translation. This would suggest that the first transmembrane helix initiates translocation and the next one causes stop-transfer, which continues in alternating fashion with the following TMDs. Although intuitively appealing, it does not seem to hold as common principle [21, 22]. In CFTR, TMH1 only interacts with Sec61 for ~ 25% of the time, while TMH2 is efficiently recognized [18]. After failed recognition, the TMH1 then must be inserted into the ER-membrane following recognition of TMH2. Which one of the translocon(s) is responsible for CFTR insertion and translocation into the ER membrane, however, remains to be defined. The nucleotide-binding domains and the R-region fold in the cytosol. The individual domains of CFTR fold mostly co-translationally, and the TMDs have been found to continue folding post-translationally, which require interactions with the other domains [7, 18]. Taken together, this supports a view that individual domains of CFTR fold mostly during translation, whereas acquisition of compactly folded domains continues post-translationally through domain–domain interactions [23].

Despite this progress, mechanistic insight into the folding pathways of CFTR domains and especially of the later assembly events into a functional structure is only understood in general terms. This is in part because transmembrane helices are embedded in a membrane and shielded from the aqueous environment, but it is also caused by the paucity of suitable reagents to study them. Here we have developed and characterized antibodies against the TMDs of CFTR. We then used them together with antibodies against the NBDs to establish an integrated workflow of radiolabeling pulse chase, limited proteolysis, and immunoprecipitation, for temporal analysis of CFTR (domain) folding in vivo. By identifying the boundaries of domain-specific proteolytic fragments, we uncovered detailed changes in conformation during maturation of newly synthesized CFTR and describe a global folding profile of the CFTR protein. We then used the assays and information to uncover folding defects in the abundant disease-causing CFTR F508del mutant as well as in mutants that had been concluded to have a defect in export from the ER.

## Results

### Characterization of antibodies against CFTR TMDs

**Fig. 1:**
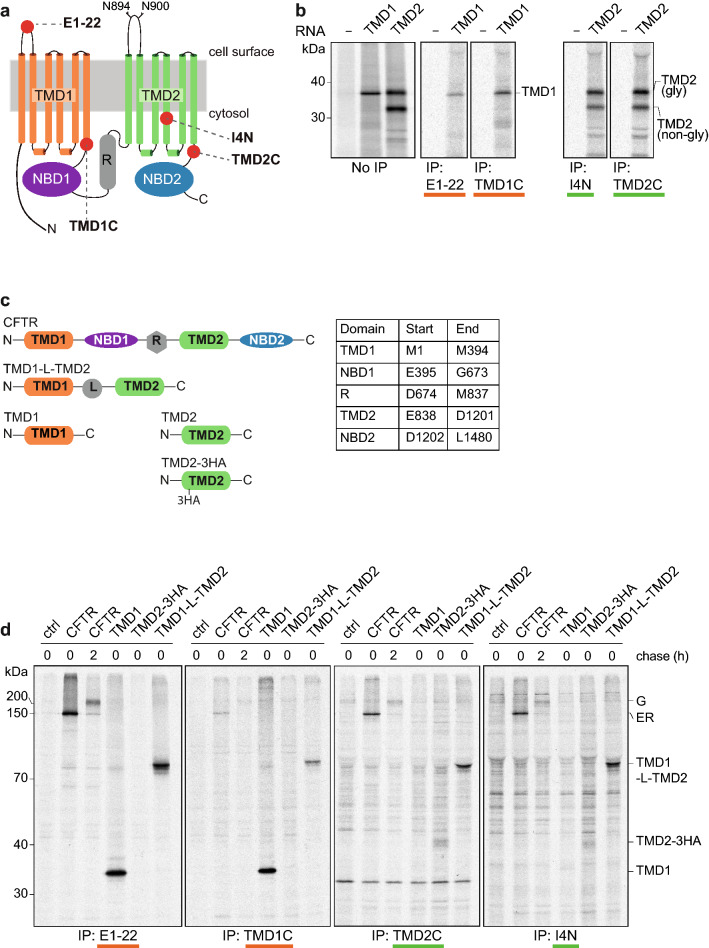
New antibodies against CFTR transmembrane domains recognize whole domains synthesized in vitro and in vivo. **a** Cartoon of CFTR in which red spheres represent the position of the epitopes to which antibodies were raised. Sites for N-linked glycosylation in TMD2 are shown. **b** TMD1 and TMD2 were translated in vitro and translocated in the presence of semi-intact cells as a source of ER membranes. Membrane fractions of in-vitro translated TMD1 and TMD2 were resolved by SDS-PAGE (left panel) or immunoprecipitated (IP) with E1-22 & TMD1C (middle), and I4N & TMD2C, respectively (right). Samples were resolved by 12% SDS-PAGE. **c** Schematic representation of constructs used in (**b**, **d**), with a table showing the used boundaries of CFTR domains. **d** HEK293T cells expressing single or multi-domain CFTR constructs were pulse labeled for 15 min and lysed immediately or after a chase of 2 h. CFTR was immunoprecipitated from detergent lysates with indicated antibodies and resolved by 10% SDS-PAGE

Limited proteolysis in combination with immune-based detection of fragments is a versatile method to assay protein folding in cells [7, 24, 25]. Most antibodies that have been available for these studies recognize exposed cytoplasmic epitopes in CFTR, which largely precludes analysis of the transmembrane domains. To extend folding studies to the transmembrane domains of CFTR, we raised antibodies in rabbits against the small first extracellular loop, peptide aa S364—K381 of TMD1, and to residues Q1035—S1049 and E1172—Q1186 of TMD2, and named them E1–22, TMD1C, I4N, and TMD2C, respectively (Fig. 1a, Table S1).

The antibodies were tested first on TMD1 and TMD2 translated in vitro in the presence of ^35^S-labeled amino acids and a source of ER membranes (Fig. 1b). TMD1 is detectable as one major protein of 35 kDa while TMD2 is resolved in bands at 32 kDa and 37 kDa. The larger form represents core-glycosylated TMD2, and the 32-kDa band is non-glycosylated TMD2 (Fig. S1a) [26]. E1–22 and TMD1C immunoprecipitated TMD1 and not TMD2, whereas I4N and TMD2C detected both forms of TMD2 but not TMD1 (Fig. 1b and not shown), showing that the antibodies recognized their target proteins in a relatively non-complex biological system, which lacks additional radiolabeled proteins.

We next asked whether the antibodies against the TMDs recognized their epitopes in intact cells. Cells expressing various single-domain and multi-domain constructs of CFTR (Fig. 1c) were labeled with ^35^S-methionine/cysteine and subsequently chased without radiolabel to follow maturation of the labeled proteins with time [27]. Newly synthesized full-length CFTR is core glycosylated and appears at the end of a 15-min pulse as a ~ 150 kDa band (Fig. S1b, MrPink, band ER) [26]. During the chase with unlabeled methionine and cysteine (2 h), CFTR is transported to the Golgi complex where the glycans are modified to complex glycans, with a concomitant increase of molecular weight to ~ 200 kDa (Fig. S1, MrPink, band Golgi) [26]. The ER form of CFTR is also called B band, and the Golgi-modified form C band.

All four antibodies recognized full-length CFTR, both the ER and Golgi forms (Fig. 1d), albeit with much lower efficiency for TMD1C and the TMD2 antibodies (I4N, TMD2C). Only E1–22 detected full-length CFTR as well as control antibody MrPink (cf. Fig. 1d with Figs. S1b, S2). Cells expressing single-domain and multi-domain constructs of CFTR were not subjected to a chase period, because these are retained in the ER and, therefore, do not reach the oligosaccharide-modifying enzymes of the Golgi complex [28]. E1–22 and TMD1C immunoprecipitated TMD1 linked to TMD2 and TMD1 expressed on its own (Fig. 1d), similar to in-vitro translated TMD1 (Fig. 1b). TMD2C and I4N detected individually expressed TMD2 but only when it contained a triple-HA tag in ECL4 or was linked to TMD1 (Fig. 1d), suggesting that TMD2 expressed on its own was not stable enough to allow accumulation of a detectable quantity. All four antibodies clearly retained their specificity in a radiolabeled cell lysate. TMD1C showed more recognition of isolated TMD1 compared to TMD1 in CFTR, suggesting shielding of the epitope for TMD1C in full-length CFTR.

### Antibodies reveal folding intermediates of CFTR

**Fig. 2:**
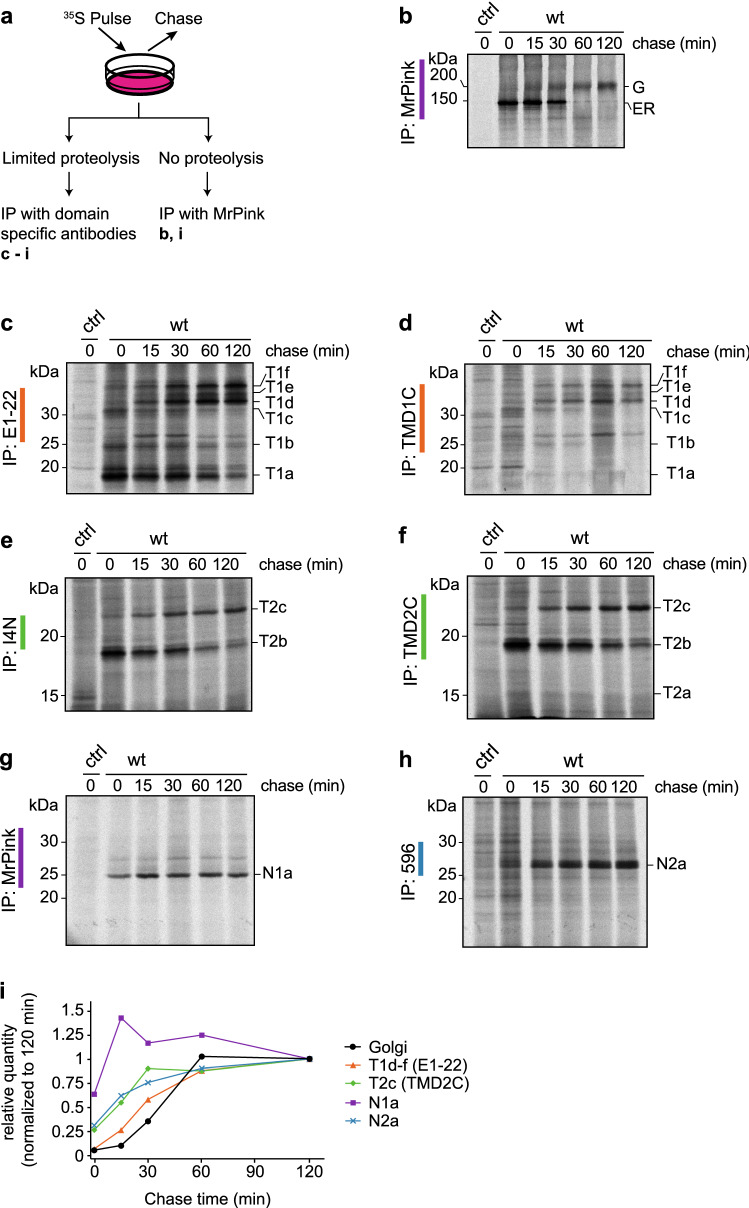
Antibodies against TMDs identify folding intermediates in vivo.** a** Workflow of radioactive pulse-chase-limited-proteolysis assay. **b** HEK293T cells expressing CFTR were pulse labeled for 15 min and chased for the indicated times. CFTR was immunoprecipitated using MrPink and immunoprecipitates were resolved by 7.5% SDS-PAGE. Remaining lysates were subjected to limited proteolysis (LP) with 25 µg/mL Proteinase K and protease-resistant fragments were immunoprecipitated with **c** E1-22, **d** TMD1C, **e** I4N, **f** TMD2C, **g** MrPink, and **h** 596 and resolved by 12% SDS-PAGE. wt, wild-type CFTR; T1a-T1f are TMD1-specific protease resistant fragments; T2a-c are TMD2-specific protease resistant fragments; N1a and N2a are protease resistant fragments specific for NBD1 and NBD2, respectively. **i** Quantification of the amount of CFTR transported to the Golgi complex and of the indicated fragments generated during limited proteolysis

We next deployed the TMD antibodies in conjunction with antibodies against the two NBDs to zoom in on the folding of individual CFTR domains in the context of the full-length CFTR protein. We used MrPink for NBD1 and monoclonal antibody 596 for NBD2 (Table S1) [24, 29]. The flexible R-region was omitted from the analysis because of its phosphorylation-dependent conformation [30]. Cells expressing full-length wild-type CFTR were radiolabeled for 15 min, and the label chased for the indicated times up to 2 h, a so-called pulse-chase protocol (Fig. 2a). CFTR was immunoprecipitated directly from detergent cell lysates using the NBD1-specific MrPink antiserum (Fig. 2a, b).

In parallel, the lysates were subjected to limited proteolysis using Proteinase K, to probe conformation of CFTR. The more folded a protein is, the more compact it becomes, and the more protease-resistant it will be. During folding, proteins acquire increasing protease resistance, detectable as an increase in proteolytic fragment size. Immunoprecipitation of the fragments with the domain-specific antibodies then allows analysis of the conformational changes in each domain of CFTR during its folding, during the chase (Fig. 2a, c–h) [7, 31].

As CFTR traveled from the ER to the Golgi complex (Fig. 2b), the proteolytic fragments immunoprecipitated by the domain-specific antibodies changed (Fig. 2c–h). Especially the TMD1 fragment profiles changed with time, from smaller fragments more prominent immediately after pulse labeling, to larger TMD1 fragments arising from CFTR after the chase. E1–22 and TMD1C immunoprecipitation detected three fragments at 0-h chase, which we named T1a, b, and c. The amounts of T1a–c decreased during the chase and three larger TMD1 fragments emerged, which we named T1d, e, and f (Fig. 2c,d).

Like the TMD1 antibodies, the TMD2 antibodies TMD2C and I4N also immunoprecipitated smaller proteolytic fragments after the pulse than after a chase. Immediately after the pulse, both antibodies detected a fragment we named T2b (Fig. 2e, f). Only TMD2C recognized an additional, smaller fragment, T2a (Fig. 2f), which was more prominent when digested from more lysate (Fig. 5a, b). After 2 h of chase, the T2b early fragment started to disappear with concomitant appearance of the larger fragment T2c (Fig. 2e, f). At that time, TMD1C, TMD2C and I4N immunoprecipitated substantial amounts of proteolyzed CFTR fragments, much more than full-length CFTR. Once fragmented, the epitopes are less shielded and, therefore, more accessible to the antibodies, implying that these three antibodies are conformation sensitive.

The NBD1-specific immunoprecipitates from the same proteolytic digests contain a major band (N1a) at ~ 25 kDa immediately after the pulse, which persists throughout the chase (Fig. 2g, i) [31–33]. Proteinase-K digestion yielded a similar single band (~ 25 kDa) from NBD2, N2a, which increased intensity during the chase (Fig. 2h, i) [31–33]. The time-dependent alterations in fragment patterns (Fig. 2i) suggested that TMD1, TMD2, and NBD2 underwent post-translational conformational changes. NBD1 folds already during synthesis [7, 24, 31] and did not follow the slow kinetics of the other domains (Fig. 2i). The increasing size of the fragments may be due to formation of more compact structures within domains or to increasing CFTR domain–domain interactions [6, 14].

We, therefore, set out to determine the identity of each proteolytic fragment. Decoding proteolytic fragments will reveal the protected areas within each domain at different stages of CFTR folding; the identification of protected and deprotected regions reports on the domain-folding and domain-assembly mechanisms. Mass spectrometry is not an option for this analysis due to the sub-picomole quantities of radiolabeled fragments in the cell lysate, leading to dearth of quantity as well as purity. Mass spec-compatible labeling procedures need labeling times corresponding to the complete maturation time of CFTR. To address the aim of fragment identification we therefore combined information on the location of the epitopes recognized by the antibodies (Table S1) and on Proteinase-K consensus cleavage sites (Table S2), with electrophoretic mobility shifts of fragments digested from CFTR truncations and point mutants, and with secondary-structure predictions.

### The CFTR N-terminus is increasingly protected post-translationally

**Fig. 3:**
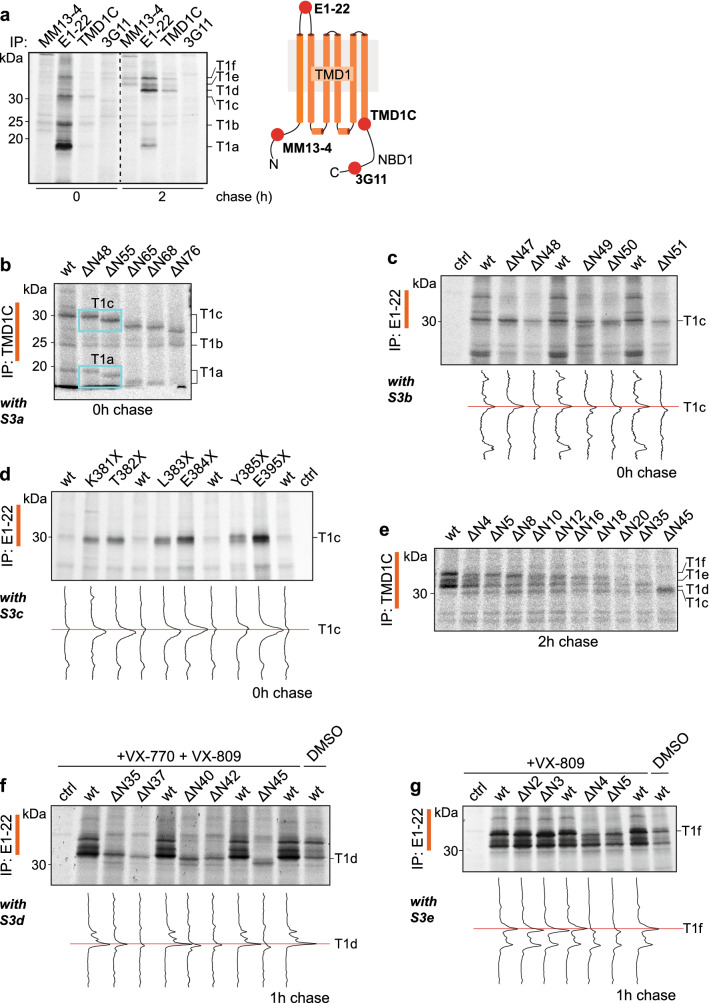
Identification of TMD1 fragments. **a** HEK293T cells expressing CFTR were pulse labeled for 15 min and lysed immediately or after a chase of 2 h. Lysates were subjected to limited proteolysis with 25 µg/mL Proteinase K and protease-resistant fragments were immunoprecipitated with indicated antibodies. Position of the antigenic epitopes in TMD1 is marked with red spheres in the cartoon on the right. **b** HEK293T cells expressing N-terminally truncated versions ΔN48 to ΔN76 of CFTR were pulse-labeled for 15 min and lysed. Lysates were subjected to limited proteolysis with 25 µg/mL Proteinase K and immunoprecipitated with TMD1C. The downward shifts of the TMD1-derived fragments are marked in cyan boxes. **c** HEK293T cells expressing N-terminal CFTR truncations ΔN47 to ΔN51 were pulse labeled for 15 min and lysed immediately. Detergent lysates were subjected to limited proteolysis with 25 µg/mL Proteinase K and immunoprecipitated with E1-22. Lane intensity profiles (ImageQuant analysis) of the fragments of interest are shown below each panel. **d** Same as (**c**) but now with C-terminal truncations K381X to E395X. **e** Same as (**b**) but now for ΔN4 to ΔN45 CFTR and after a chase of 2 h. **f** Same as (**c**) with N-terminal truncations ΔN35 to ΔN45, but the 15-min pulse labeling was followed by a 1-h chase. **g** Same as (**f**) but now with N-terminal truncations ΔN2 to ΔN5. Experiments in panels (**f**, **g**) were done in the presence of VX-770 (3 µM) and/or VX-809 (3 µM) as indicated. The undigested samples corresponding to panels b–d and f–g are in Fig. S3a–e. All samples were resolved by 12% SDS-PAGE. *wt* wild-type CFTR

All fragments from the TMD1-specific immunoprecipitates contained the epitope recognized by E1-22 (aa A107—S118) in ECL1, but only the larger T1e and T1f fragments were detected by MM13-4 (aa G27—L34) (Fig. 3a, 2-h chase). This showed that the N-terminal boundaries of proteolytic TMD1-derived fragments T1e and T1f must have been *upstream* of the epitope of MM13-4, between residues M1 and G27. The N-terminal boundaries of T1a-d however must be *downstream* of L34, between epitopes seen by MM13-4 and E1-22, i.e. between residues L34 and A107. To zoom in, we generated 24 N-terminally truncated versions of CFTR from the far N-terminus (∆N2) to the first transmembrane helix (∆N76). When the deleted residues are part of a fragment, that fragment should be truncated too and shift down in the gel. Constructs were expressed in HEK293T cells and analyzed by SDS-PAGE for the impact of truncations on the patterns of proteolytic TMD1 fragments at 0-h, 1-h, or 2-h chase times (Figs. 3 and S3). Precise positions of peaks were compared by line scans of gel lanes. This procedure allowed identification of the N-termini of the individual fragments.

The size of the T1c and T1a proteolytic fragments decreased when derived from CFTR lacking 55 (ΔN55) or more N-terminal residues, but not yet after truncating 48 residues, ΔN48 (Fig. 3b), indicating that the N-termini of T1c and T1a arose from proteolysis between residues N48 and R55. The parallel and gradual decrease of T1a and T1c upon further truncation (Fig. 3b) suggested that T1a and T1c share their N-termini. A deletion series of D47 to E51 (Figs. 3c and S3a) confirmed Leu49 as the N-terminal cleavage site for T1a [31] and for T1c.

To find the C-terminal boundary of T1c, we used the same strategy, but now with C-terminal truncations of CFTR. Introducing a stop codon at positions E384, Y385, or N386 close to the C-terminus of TMD1 (aa M394) did not change T1c mobility, whereas shorter constructs (truncated at K381, T382, or L383) did (Fig. 3d). T1c thus represents fragment S50-L383, with Leu49 and Leu383 as the most prominent protease cleavage sites. We had defined fragment T1a as aa S50–S256 [31], which is consistent with T1a lacking the epitope of TMD1C (aa S364–K381). Yet, T1a at times does appear in TMD1C immunoprecipitations (Fig. 3b), suggesting that despite the proteolytic cleavage at Ser256 in ICL2, the N-terminal and C-terminal halves of TMD1 stay tightly associated upon non-denaturing cell lysis. Cryo-EM structures [14, 16–18] and structural models [13, 15] confirm that in native CFTR, TMH5 and TMH6 interact with residues in TMH1-4.

T1b must be a mixture of at least two separate, mutually exclusive fragments. T1b appears in immunoprecipitations with MM13-4 (epitope aa G27–L34) and also with TMD1C (epitope aa S364–K381) (Fig. 3a, b) and is too small to contain both epitopes. Indeed, the T1b fragment recognized by TMD1C does not change mobility when CFTR is truncated up to 76 residues from the N-terminus (Fig. 3b). Because the fragment was not unique, we did not invest in its identification.

To identify the C-terminal boundaries of the later-appearing fragments T1d–f, C-terminally truncated constructs cannot be used, as TMD1 requires downstream domains to reach the fully native fold [28] and hence does not acquire sufficient protease resistance to yield T1d–f when truncated. We, therefore, relied on results of epitope mapping to determine the C-terminus (Fig. 3a). T1d–f was immunoprecipitated by TMD1C (epitope S364–K381 at the C-terminus of TMD1), but not by the 3G11 antibody (epitope N396–F405 at the N-terminus of NBD1). We concluded that the C-terminal boundaries of T1d-f are in the linker region between TMD1 and NBD1 (Fig. 3a). Proteinase-K consensus sites in this region are L383, L387 and M394. The C-terminus of T1c arises from cleavage at Leu383. Because T1c results from digestion of less packed and more open forms of CFTR, the early folding and domain assembly intermediates, Leu383 is the more likely cleavage site compared to M394 and L387. Moreover, M394 is located in a β-sheet [14] and is less likely to be cleaved. We concluded that Leu383 is the most probable cleavage site and last residue of not only T1c but also fragments T1d-f.

To identify the N-termini of fragments T1d–f, we again expressed N-terminally truncated versions of CFTR, as done above for T1a and T1c. Proteolytic digestion and immunoprecipitation with the TMD1C antiserum showed that deletion of 4 to 45 residues increased the electrophoretic mobilities of T1f, T1e, and T1d sequentially (Fig. 3e), implying that these three fragments differed in their N-termini. Because T1d–f only arise late in biosynthesis, their analysis requires a chase period, during which many truncated constructs were degraded (Fig. 3e). To facilitate analysis of fragments T1d–f, we added corrector and potentiator compounds VX-809 and VX-770 [34–38]. VX-809 stabilizes TMD1 and thereby enhances expression of almost all CFTR mutants [34–38]. VX-770 destabilizes T1f at the gain of T1d, improving detection of T1d [25, 39]. The T1d fragment was immunoprecipitated with TMD1C (epitope aa S364–K381) lacking 35 (∆N35), but not 45 residues (∆N45) from the N-terminus, (Fig. 3e). The N-terminus of T1d hence lies between aa S35 and S45, consistent with the lack of detection by MM13-4 antibody (epitope aa G27–L34) (Fig. 3a, right panel). Truncation analysis of aa S35–––S45 revealed that deleting 37 residues (∆N37) prevented immunoprecipitation with E1-22 (epitope aa A107–S118) whereas removing 35 residues (∆N35) still allowed detection of T1d (Fig. 3f). T1d thus starts from aa D36, with Ser35 being the most prominent protease cleavage site.

The signal of T1e was not strong in wild-type CFTR, and removing residues from the N-terminus eventually caused collapse of T1f onto T1e (Fig. 3e). As T1e was immunoprecipitated by MM13-4 (epitope aa G27–L34) (Fig. 3a, right panel), we concluded that the N-terminus of T1e lies between residues W19 and K26 in the Lasso helix 1 (Lh1). T1e was present in digests from CFTR lacking the first 16 or 18 amino acids, was only a very fuzzy fragment from the ∆N20 deletion and, as expected, absent upon truncation at amino acid S35 (Fig. 3e). The most likely boundary of T1f is residue F17 or S18, from cleavage after Phe16 or Phe17. Yet, the fuzzy nature of the T1e band and its frequent absence—such as from ∆N5 (Fig. 3e)— are consistent with the dynamic nature of Lh1 and its embedding in the membrane [14, 15, 40].

T1f had already decreased after truncation of only 4 amino acids of CFTR (ΔN4, Fig. 3e). Upon closer inspection, even deleting the first two amino acids (ΔN2, Fig. 3g) resulted in a downward shift and we conclude that T1f starts from the first amino acid of CFTR.

**Fig. 4:**
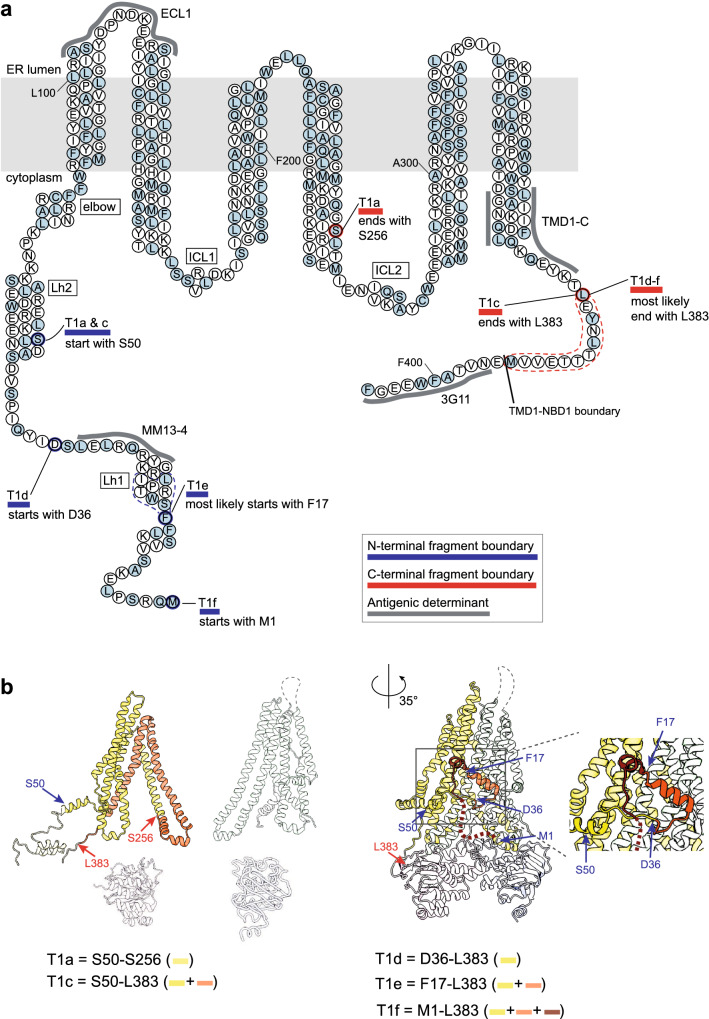
Summary map of TMD1 fragments.** a** TMD1 amino-acid sequence highlighting the Proteinase-K cleavage sites that result in TMD1 fragments T1a–T1f. All alpha helices are depicted as columns of 3 residues wide, with the exception of ICL1, which includes a coupling helix of only 5 residues (SRVLD). T1a extends from aa S50 to S256, T1c from S50 to L383, T1d from D36 to L383, T1e from F17 to L383, and T1f from M1 to L383. Key: light blue circles: Proteinase-K consensus cleavage residues; grey lines: antigenic epitopes; blue lines: N-terminal boundaries of fragments; red lines: C-terminal boundaries of fragments; dotted lines: possible cleavage area; Lh1: location of Lasso helix 1; Lh2, location of Lasso helix 2; elbow: location of the N-terminal elbow helix. **b** TMD1 proteolytic fragments in structure representation. The left panel shows fragments T1a and T1c with their fragment boundaries. The structure is based on CFTR models with the N-terminus in the cytoplasm [13, 15] because this better encapsulates the conformation of the protein during vectorial folding, before the domains have assembled; the cryo-EM structures represent the mature, domain-assembled form, which has not yet been reached. The right panel shows fragments T1d-f with their fragment boundaries. For this panel we used the cryo-EM structure (PDB: 5UAK) [14], as T1d-f represent domain-assembled, mature CFTR

Results on identification of TMD1 fragments are compiled in Tables 1 and S3 and shown in Fig. 4. In summary, T1a starts from N-terminal Lasso helix 2 (Ser50), includes ICL1 and TMH1-4, and ends at Ser256, halfway down the descending ICL2 helix extending from TMH4 [31]. T1c has the same N-terminus as T1a but is protease-protected at the C-terminus of T1a, contains both ICL1 and ICL2, and ends at Leu383 in the linker between TMD1 and NBD1. The difference between the early T1a and T1c fragments and the late T1d–f fragments is the N-terminal region upstream of Lh2, a 50-amino acid stretch that includes Lasso helix 1 and the ultimate N-terminus of CFTR (Fig. 4, Table 1). While the protein folds, the N-terminus of CFTR becomes more protected and resistant to protease.

**Table 1:** Summary of fragment identities

Fragment	Residue boundaries	Structural boundaries	Residue boundaries	Structural boundaries
	N		C	
T1a	S50	Lh2	S256	TM4, N-terminal of ICL2
T1c			L383 (T1d-f; 383–394)	Linker TMD1 to NBD1
T1d	D36	Loop between Lh1 and Lh2		
T1e	F17 (17–24)	Lh1		
T1f	M1	Start CFTR		
T2a	K1060	End of TM10, start of ICL4	M1191 (T2c; 1187–1196)	Linker TMD2 to NBD2
T2b	N965	ICL3, middle of coupling helix		
T2c	T910	End of ECL4, start of TM8		
N1a	L428	In RI region	F653	In RE region
N2a	M1191	Linker TMD2 to NBD2	Q1439	C-terminus of NBD2

Summary of residue boundaries of the proteolytic fragments and their locations relative to the structure of CFTR. Where the boundaries are defined by ranges of amino acids, the most likely residue is followed by the range in brackets

### ICL4 in TMD2 is protected during post-translational folding

**Fig. 5:**
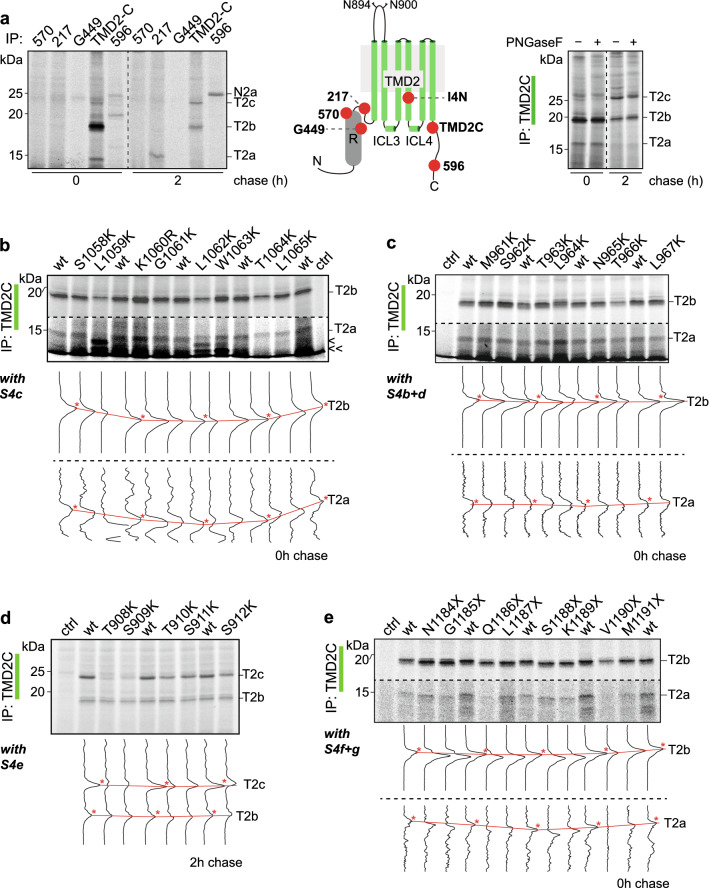
Identification of TMD2 fragments.** a** HEK293T cells expressing CFTR were pulse labeled for 15 min and lysed immediately or after a chase of 2 h (left panel). Lysates were subjected to limited proteolysis with 25 µg/mL Proteinase K and protease-resistant fragments were immunoprecipitated with indicated antibodies. Position of the antigenic epitopes in TMD2 is marked with red spheres in the cartoon (middle panel). The right panel shows TMD2-fragment immunoprecipitates treated with PNGaseF. **b** HEK293T cells expressing CFTR mutants S1058K to L1065K were pulse labeled for 15 min and lysed immediately. **c** Same as (**b**) but with mutants M961K to L967K. **d** same as (**b**) with mutants T908K to S912K, but now after a chase of 2 h. **e** Same as (**b**) with C-terminal truncations N1184X to M1191X. Detergent cell lysates subjected to limited proteolysis with 25 µg/mL Proteinase K were immunoprecipitated with TMD2C. Samples were resolved by 12% SDS-PAGE. Lane intensity profiles (ImageQuant analysis) of the fragments of interest are shown below each panel. The undigested samples corresponding to panels (**b**–**e**) are in Fig. S4c–f. *Wt* wild-type CFTR, T2a, T2b, and T2c are TMD2-specific protease-resistant fragments. Red asterisks mark the wild-type peaks between which straight lines were drawn for reference

Our strategy to determine the N-terminal boundaries of the TMD2 fragments started with immunoprecipitations using the antibodies 217, 570, and G450 against R (Fig. 5a, left and middle panels, and Table S1), which is directly N-terminal of TMD2. None of these brought down TMD2 fragments, showing that the fragments do not include large parts of R (Fig. 5a, left panel). The fourth extracellular loop (ECL4) in TMD2 contains N-linked glycans at amino-acid residues N894 and N900. We then interrogated TMD2C immunoprecipitates of limited-proteolysis fragments for the presence of N-linked glycans using PNGaseF digestion. In contrast to full-length CFTR (Figure S2), none of the proteolytic fragments shifted downwards in the gel following the PNGaseF glycanase treatment (Fig. 5a, right panel), showing that the TMD2 fragments do not contain residues N894 and N900. Because T2a was immunoprecipitated using TMD2C (epitope E1172 –Q1186), but not I4N (epitope Q1035– S1049), we place the N-terminal boundary of T2a after TMH10 (Figs. 5a and 2e–f).

Generating truncations at the N- or C-termini of TMD2 not only disturbs the interactions with other domains, but also may affect early folding events [28, 29]. For precision mapping the N-terminal boundaries of TMD2 fragments, we, therefore, used an alternative strategy than deletion analysis. Electrophoretic mobility of proteins in SDS-PAGE often deviates from the predicted mass [41], and especially small polypeptides may be affected by their charge [42]. We found that changing residues of CFTR ICLs into lysine indeed affected mobilities of proteolytic TMD2 fragments, which provided a starting point for investigating the N-terminal boundaries of the proteolytic fragments T2a and T2b using site-directed mutagenesis.

To determine the N-terminal boundary of T2a, we performed a lysine scan on ICL4, mutating one residue at a time to a lysine and subjecting to pulse-chase, limited proteolysis and immunoprecipitation (Fig. 5b). The T2a fragment shifted slightly upwards when residues G1061–T1064 were mutated to a lysine, and did not shift in S1058K nor K1060R. This implies that residues G1061–T1064 were part of the T2a fragment, and S1058 and K1060R perhaps not. A Lys-to-Arg mutation, however, is not expected to change mobility, because the charge did not change and SDS binding was likely the same. We concluded that Leu1059 must be the cleavage site that generates T2a, which then starts with K1060. This was underscored by the phenotype of the L1059K mutant, which then should remove that cleavage site: L1059K indeed did not yield T2a, but two smaller fragments instead (Fig. 5b, < and < <). Similar albeit not identical patterns emerged from L1062K and W1063K. The small bands appeared at the expense of T2b in the L1059 and L1062 mutants, while radiolabeled full-length protein levels were the same (Figure S4c). These mutations must have changed CFTR conformation and exposed otherwise protected proteolytic sites, likely including L1062 and W1063.

T2b is larger than T2a (which starts with K1060) and was recognized by I4N (epitope aa Q1035–S1049) (Fig. 2e). This placed the N-terminal boundary of the fragment upstream of residue Q1035, with ICL3 being the most accessible region for the protease [14]. We, therefore, mutated residues in ICL3 to lysine, one at a time, and analyzed whether the mutation shifted the T2b fragment on gel (Fig. 5c), as done for T2a above. We examined lysine mutants of residues L957 to G970 (Figs. 5c and S4b, d) and found L964K T2b to shift upwards significantly. The L964K mutation likely eliminated the preferred Proteinase-K cleavage site to generate T2b, perhaps to the benefit of S962, as this would explain the upshift of T2b from L964K. Electrophoretic mobility of T2b appears less sensitive to charge mutations than the other fragments because mutants between L964 and G970 did not cause a mobility shift. We nonetheless conclude that T2b starts with N965 upon Proteinase-K cleavage after L964.

As T2c did not contain glycans (Fig. 5a) and had a higher mass than T2b (which starts with N965), we hypothesized that the N-terminal boundary was in ECL4 downstream of the N-linked glycosylation sites. Residues C-terminal to the N-glycosylation site N900 in ECL4 were mutated to lysine to identify the N-terminal boundary of T2c (Figs. 5d and S4a, e). Mutants N901K to V905K yielded T2c with unchanged mobility (Fig. S4a), which eliminated Proteinase-K consensus sites S902 and A904 as possible T2c protease cleavage sites and suggested that these residues were not part of T2c. In contrast, lysine mutations in residues S911 and S912 did decrease mobility, implying that they were included in T2c. In mutants T908K and S909K, between the non-affected (N901–V905) and affected residues (S911, S912), the intensity of T2c was decreased significantly. As threonines are not favored Proteinase-K cleavage sites, we conclude that Ser909 is the most likely cleavage site to generate T2c, positioning its N-terminal boundary at Thr910.

**Fig. 6:**
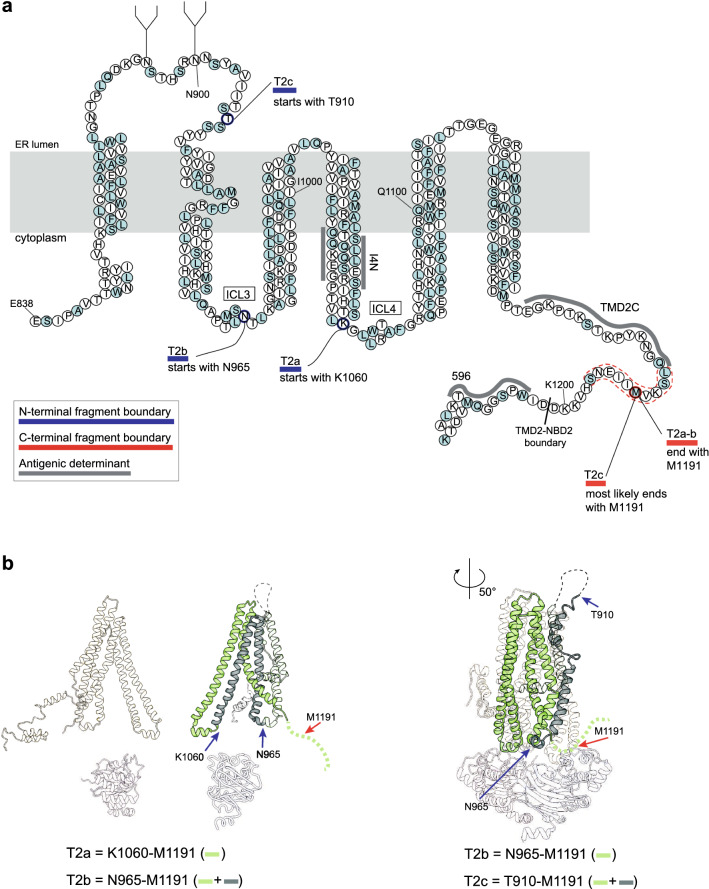
Summary map of TMD2 fragments.** a** Representation of the TMD2 amino-acid sequence highlighting the Proteinase-K cleavage sites that result in TMD2 fragments T2a-T2c. All alpha helices are depicted as columns of 3 residues wide. T2a extends from aa K1060 to M1191, T2b from N965 to M1191 and T2c from T910 to M1191. Key: light blue circles: Proteinase-K consensus cleavage residues; grey lines: antigenic epitopes; blue lines: N-terminal boundaries of fragments; red lines: C-terminal boundaries of fragments; dotted lines: possible cleavage area. **b** TMD2 proteolytic fragments in structure representation. The left panel shows fragments T2a and T2b with their fragment boundaries. As in Fig. 4b, the structure is based on CFTR models with the N-terminus in the cytoplasm [13, 15] because this better encapsulates the conformation of the protein during vectorial folding, before the domains have assembled; the cryo-EM structures represent the mature, domain-assembled form, which has not yet been reached. The right panel shows fragment T2c with its fragment boundaries. For this panel we did use the cryo-EM structure (PDB: 5UAK) [14], as T2c represents domain-assembled, mature CFTR

By analyzing the presence of antigenic epitopes on the fragments, we conclude that the C-terminal boundaries for the TMD2 fragments were between aa K1189 – I1203, as they all were immunoprecipitated by TMD2C (epitope E1172–Q1186) but not 596 (epitope W1204–T1211) (Fig. 5a, left and middle panels). Replacing V1190 with a stop codon still generated T2b, whereas shorter constructs led to a downward shift (Figs. 5e and S4f, g). The shift changes were also seen for T2a, which would suggest that the last residue of both fragments would be K1189. This is not likely as K is not cleaved by Proteinase K. More probable is M1191 as preferred cleavage site and C-terminal residue of the TMD2 fragments, because it is the first cleavable residue upstream of K1189. The aberrant mobilities of the fragments derived from M1191X and V1190X may well be caused by the positive charge of the lysine residue close to their C-terminus. Removal of the K in K1189X leads to an immediate shift down in the gel. Indeed, if anything, V1190X 'T2b' may run even slightly higher than wild-type T2b. We, therefore, concluded that T2a consists of K1060–M1191, whereas T2b is N965–M1191. The C-terminus of T2c could not be determined this way as it was not detected in C-terminally truncated CFTR after the chase (data not shown). We nonetheless concluded, considering the epitopes, that T2c most likely is cleaved at M1191 as well and encompasses T910 – M1191 (Fig. 6).

Results on identification of TMD2 fragments are compiled in Table S4. In summary, T2a starts near the beginning of ICL4 until the end of TMD2. T2b shares its C-terminus with T2a, but starts at the N-terminus within ICL3. With time, T2c appears and increases; this fragment starts at the C-terminal end of ECL4 (Fig. 6, Table 1). The change from T2a–b to T2b–c shows that ICL4 becomes protected while CFTR folds.

### N1a represents NBD1 cleaved in regulatory insertion and extension

**Fig. 7:**
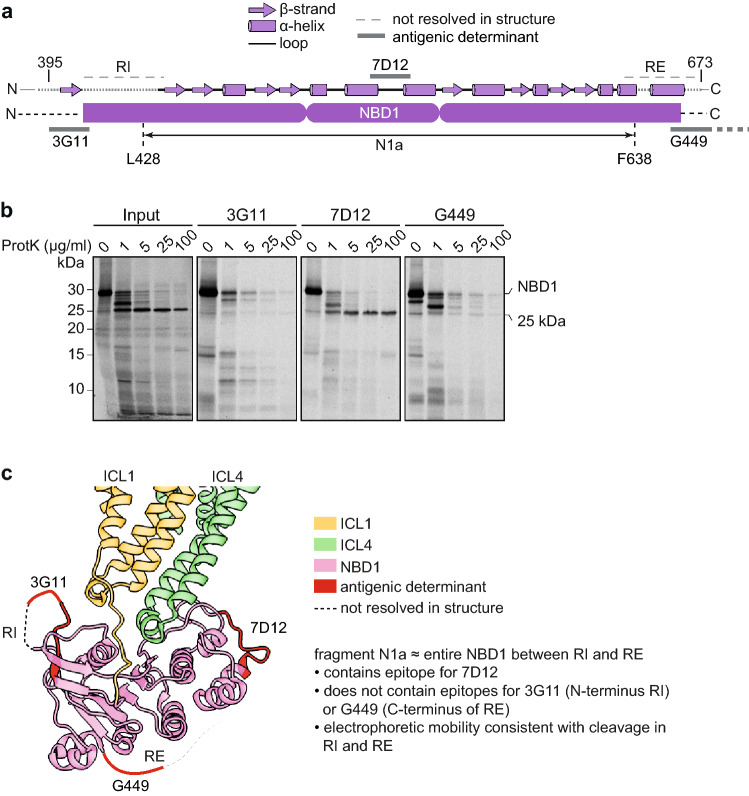
The N1a fragment represents NBD1 without RE and RI.** a** Secondary structure of NBD1 including Regulatory Insertion (RI) and C-terminal Regulatory Extension (RE), with antigenic epitopes indicated and the identity of the N1a fragment shown, resulting from cleavages in RI and RE, likely from residues L428 to F653. **b** NBD1 was translated in vitro at 30 °C for 30 min and proteolyzed on ice with different concentrations of Proteinase K; immunoprecipitations were done with the indicated antibodies. Full-length NBD1, 27-kDa and 25-kDa fragments are indicated. **c** Structure of NBD1 and interacting TMD elements ICL1 and ICL4 (PDB: 5UAK) [14] indicating the antigenic epitopes in red. N1a contains NBD1 without most of RI (as well as upstream sequence) and RE

The nucleotide-binding domains both yielded a major ~ 25-kDa protease-resistant fragment, which we named N1a and N2a (Fig. 2g, h) [24]. To map the boundaries of N1a, we produced NBD1 by in vitro translation, subjected the domain to limited proteolysis with Proteinase K, and immunoprecipitated fragments with antibodies recognizing distinct epitopes on NBD1 (Fig. 7 and Table S1). The N1a fragment contained the 7D12 epitope (531– 540) in the α-helical subdomain, but lacked the distal N- and C- terminal epitopes for the 3G11 antibody (396 – 405) and G449 antibody (against R-region residues 693 – 716), respectively (Fig. 7a–c) [24]. This implied that N1a must have been smaller than residues 406 – 693, as this would translate to ~ 32 kDa. This region contains at its N-terminus the disordered Regulatory Insertion (RI), and at its C-terminus the less ordered Regulatory Extension (RE), which each contain > 10 Proteinase-K cleavage sites. Taken together, we concluded that the N1a fragment contains almost complete NBD1 and was formed by cleavage inside the relatively unstructured Regulatory Insertion and Regulatory Extension, which leads to loss of both N- and C-termini (Fig. 7a,b and Table S5).

### N2a contains almost entire NBD2

**Fig. 8:**
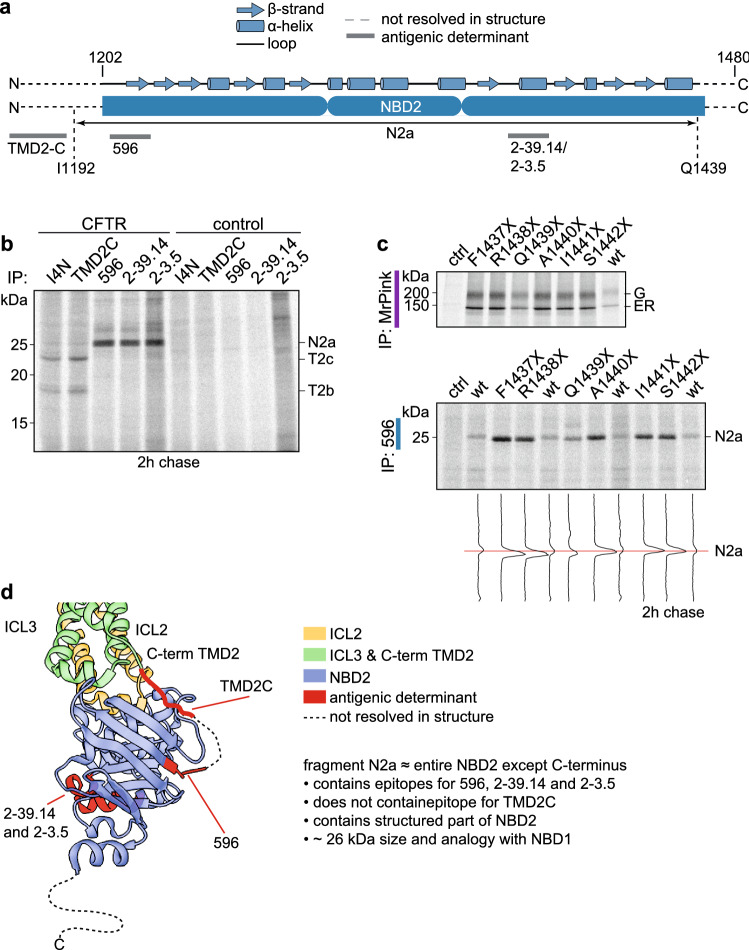
The late NBD2 fragment contains almost all of NBD2. **a** Secondary structure of NBD2 with antigenic epitopes indicated and showing the identity of the N2a fragment, residues I1192-Q1439. **b** HEK293T cells expressing CFTR or not (control) were pulse labeled for 10 min and chased for 2 h. After digestion with 25 µg/mL Proteinase K, proteolytic fragments were immunoprecipitated in parallel with I4N, TMD2C, 596, 2–39.14, and 2-3.5 antibodies and resolved by 12% SDS-PAGE. **c** HEK293T cells expressing CFTR or not (control) were pulse labeled for 10 min and chased for 2 h. After digestion with 25 µg/mL Proteinase K, proteolytic fragments were immunoprecipitated in parallel with I4N, TMD2C, 596, 2–39.14, and 2-3.5 antibodies and resolved by 12% SDS-PAGE. **d** HEK293T cells expressing C-terminally truncated CFTR constructs F1437X to S1442X were pulse-labeled for 15 min and chased for 1 h and subsequently digested or not with 25 µg/mL Proteinase K for 15 min on ice. Non-digested lysates (upper panel) were immunoprecipitated with MrPink and proteolyzed samples with NBD2-specific antibody 596 (lower panel) and resolved by 7.5% or 12% SDS-PAGE, respectively. Lane intensity profiles (ImageQuant analysis) of N2a are shown below the panel. **e** Structure of NBD2 and interacting TMD elements ICL2 and ICL3 (PDB: 5UAK) [14] indicating the antigenic epitopes in red. Dotted lines denote unresolved residues in the structure

To determine the N-terminus of N2a, we analyzed the presence of antigenic epitopes on the fragment (Fig. 8a–c). As shown in Fig. 8b, N2a was immunoprecipitated by 596 (epitope aa W1204–T1211), 2–39.14 (epitope aa E1371–R1385) and 2–3.5 (epitope aa V1379 –T1387) but not by TMD2C (epitope aa E1172–Q1186). We, therefore, conclude that the N-terminus of N2a was in the linker region of TMD2 and NBD2 (aa K1189–I1203). We conclude that M1191, which is the C-terminal cleavage site of all three TMD2 fragments, is the logical N-terminal boundary of N2a, because the linker has only few Proteinase-K consensus sites, M1191, S1196, and perhaps W1204. W1204 is blocked by the proline in 1205 and we cannot exclude that S1196 remains exposed to protease in folded, domain-assembled CFTR and the N2a-yielding cleavage is at S1196.

The C-terminal region of NBD2 (F1437-L1480) is not resolved in the cryo-EM structure [14], and is thought to be disordered. Using the C-terminal-truncation strategy used above for T1a,c, we found that replacing A1440 with a stop codon still immunoprecipitated N2a, whereas shorter constructs led to a downward shift (Fig. 8c, bottom panel). N2a hence encompasses complete NBD2 except the C-terminus, from the linker between TMD2 and NBD2 to residue A1440 (Fig. 8d, Table S6).

### Mutations in the diacidic ER export motif cause a global folding defect in CFTR

**Fig. 9:**
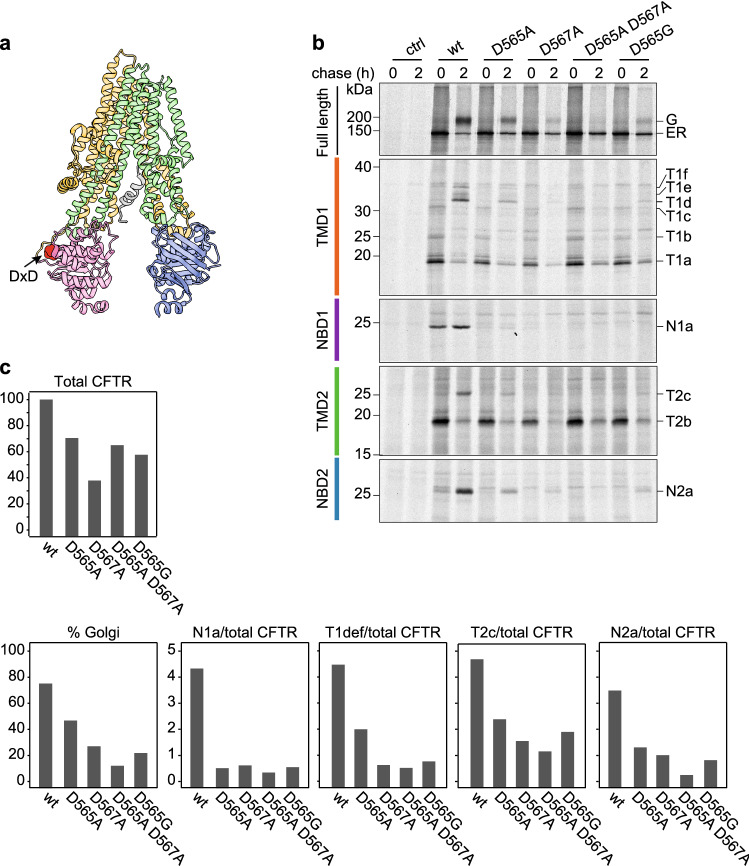
Folding analysis of CFTR mutated in an NBD1 di-acidic ER-export motif.** a** Structure of CFTR showing the location of the di-acidic ER exit motif (arrow & red-shaded area; PDB: 5UAK) [14]. **b** HEK293T cells expressing CFTR variants were pulse-labeled for 15 min and lysed immediately or after a chase of 2 h. CFTR was immunoprecipitated from non-proteolyzed lysate with MrPink and resolved by 7.5% SDS-PAGE. Proteolysis was done with 25 µg/mL Proteinase K and domain-specific fragments were immunoprecipitated with E1-22 (TMD1), MrPink (NBD1), TMD2C (TMD2), and 596 (NBD2) and analyzed by 12% SDS-PAGE. **c** Quantitation of total amounts of wild-type and mutant CFTR at 2-h chase time, of the % that had been transported to the Golgi complex and of the T1def (TMD1), T2c (TMD2), and N2a (NBD2) fragments, as measure of domain assembly

Having characterized the antibodies and the biochemical folding process, we set out to investigate the effect of mutations in the diacidic (DxD) motif in NBD1 (D565-A566-D567; Fig. 9a) on the folding of CFTR. Substitution of Asp567 to alanine impairs Sec24 binding and COPII dependent export of CFTR from the ER [43, 44]. It is not clear, however, whether these phenotypes are a direct consequence of the mutation in the interaction motif or rather are secondary and reflect a primary defect in folding of NBD1 and assembly of full-length CFTR. Such a difference is suggested by the different (chaperone) interactome of the D565A-D567A mutant compared to that of wt CFTR [45]. We created alanine mutations for each of the aspartate residues and made a construct for the D565G patient mutation [46], and determined the effects on CFTR folding using the combined tools and information in a radioactive pulse chase-limited proteolysis-immunoprecipitation assay.

As shown in Fig. 9b (top panel) and 9c (%Golgi), the acquisition of the complex glycosylated form was impaired by mutations in D565 and D567. The effect of the D567 mutation was stronger than those of D565 even though a change in the latter is associated with CF [47, 48]. Of note for each of the mutants is that the early steps in the folding path of NBD1 were impaired already because the N1a fragment was barely detectable after the pulse period. In accordance with the notion that NBD1 folding is a limiting step in CFTR biogenesis [20, 49, 50], we found deleterious effects on the amounts of late TMD1, TMD2, and NBD2 fragments at the 2-h chase time, albeit slightly milder for the D565A mutant (Fig. 9b, c). The double mutant and the patient mutant D565G were degraded less (Fig. 9c, Total CFTR) than expected on the basis of their decreased transport to the Golgi complex (Fig. 9c, %Golgi). This increased stability was not due to any improved folding. The domain-assembly-representing fragments T1def, T2c, and N2a (Fig. 9c) correlate well with transport to the Golgi complex, implying that the ER did not retain a folded pool of DXD mutant protein. Our data demonstrate that the ER-export defects of the DxD-mutants were explained in full by their folding defects.

Application of the new antibodies raised against the TMDs thus has demonstrated that mutations in the diacidic export motif cause misfolding of CFTR into an F508del-like phenotype: misfolding of NBD1 and as a result no assembly with the other domains [25, 32, 39]. Investigations into the interactions of CFTR with the COPII sorting machinery (and other relevant interactors) using mutational approaches thus need to be complemented with careful analysis of the folding status of these mutants.

### Correctors boost domain assembly without correcting NBD1 folding

**Fig. 10:**
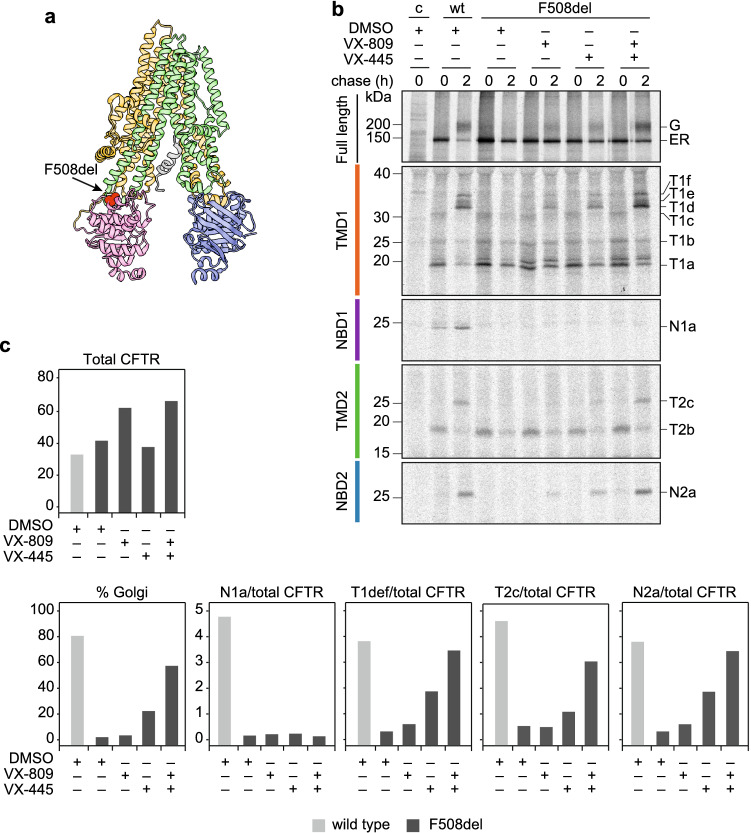
Correctors boost F508del CFTR domain assembly without correcting underlying defect in NBD1. **a** Structure of CFTR showing the location of F508del (arrow & red-shaded area; PDB: 5UAK) [14]. **b** HEK293T cells expressing wt CFTR and F508del CFTR were treated with corrector compounds VX-809, VX-445 or both (at 3 µM final concentration) during starvation, labeling and chase, and subsequently processed as in Fig. 9b. **c** Quantitation of wt CFTR and F508del CFTR transported to the Golgi complex and of domain assembly as the amount of fragment corrected for total amount of CFTR

Finally, we used the coupled radiolabel-pulse chase-limited proteolysis- immunoprecipitation folding assay to assess whether we could interrogate the influence of CFTR modulators on the folding pathway of the most prevalent F508del CFTR mutant (Fig. 10a). We focused on the type-1 corrector compound VX-809 and the novel VX-445 corrector that at least additively –via complementary modes of action– improve CFTR maturation [51]. In control cells, ~ 80% of newly synthesized wild-type CFTR molecules reached the Golgi complex within 2 h, whereas F508del CFTR did not acquire the complex glycosylated phenotype (Fig. 10b, c). In the presence of 3 µM VX-809, F508del CFTR reached the Golgi complex to 5% of wild-type CFTR (close to detection level), while this had increased to 30% in the presence of 3 µM VX-445 and even 70% in the presence of both compounds. Whereas VX-809 increases CFTR quantity (i.e. stability) (Fig. 10b, c; Total CFTR [31], VX-445 increased transport to the Golgi (Fig. 10b, c; %Golgi), consistent with their different modes of action, and thus their complementarity. In the limited-proteolysis assay, we already found appreciably improved assembly of F508del CFTR after 2 h of chase in the presence of the individual correctors, as seen by the increase in signal for the T1def fragments originating from TMD1 (orange), T2c from TMD2 (green), and N2a (blue) from NBD2, compared to the control situation for F508del CFTR without drugs (Fig. 10b, c). When cells were treated with the combination of both drugs, the amounts of these fragments increased to levels that correlated well with the % that had left the ER and reached the Golgi complex (Fig. 10b, c). Rescue of F508del CFTR by the drug combination hence led to improved domain assembly, stage 2 of the CFTR folding process. Striking is the conspicuous absence of any sign of improvement of NBD1, stage 1 (Fig. 10b, c; N1a panel). Even though F508del CFTR in the presence of both corrector compounds was transported to the Golgi complex to 70% of wild-type CFTR, we did not detect a corresponding co-increase in the amount of N1a fragment, showing that F508del NBD1 remained unfolded.

In conclusion, notwithstanding the lack of correction of F508del-NBD1 folding by the two correctors, especially the combination of VX-809 and VX-445 showed a dramatic improvement on domain assembly and, as a consequence, export of F508del CFTR to the Golgi complex. Importantly, we now demonstrate that corrector-enhanced domain assembly can compensate for the detrimental folding defect the F508del mutation causes first in NBD1 and then in its assembly with other domains into a functional channel.

## Discussion

**Fig. 11:**
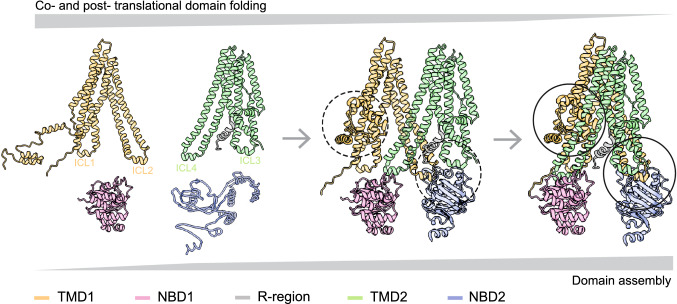
Two-stage folding process of CFTR. The left structure illustrates stage 1 of folding: TMD1, NBD1, and TMD2 fold already co-translationally and acquire a structure as if expressed on their own [7, 31]. ICL1 docks onto NBD1 already during synthesis [31], whereas the N-terminus of TMD1 cannot have native structure yet and may hang off the ER membrane [13] and/or associate with NBD1 [15]. NBD1 reaches its native protease-resistant fold completely co-translationally, which does not change upon domain assembly. Structure is based on CFTR models with the N-terminus in the cytoplasm [13, 15]. The middle structure shows a putative domain-assembly intermediate, in which the domain interfaces form fast and cooperatively, yielding the right structure. The fully domain-assembled CFTR (right structure) has ICL1 of TMD1 and ICL4 of TMD2 docked onto NBD1. The TMDs assemble, such that ICL2 of TMD1 and ICL3 of TMD3 associate with and stabilize NBD2. The circles show the sites that gain significant protease resistance around the time CFTR leaves the ER, reaches the Golgi complex and obtains complex glycans. ICL2, ICL3, and NBD2 acquire increased protease resistance simultaneously (right circle), as does the N-terminus of TMD1, which becomes protease resistant by wrapping around TMD1 and TMD2 (left circle). Structure in middle and right panels are based on cryo-EM structure (PDB: 5UAK) [14]

The folding of multi-domain, polytopic membrane proteins such as ABC-transporters is complex. In this study, we raised antibodies to the transmembrane domains of CFTR, which recognized their epitopes in full-length CFTR and proteolytic fragments arising from limited digestion with Proteinase K. Using these antibodies together with previously developed antibodies against the nucleotide-binding domains, we identified domain-specific proteolytic fragments and present the 2-stage folding pathway of CFTR in cells (Fig. 11). During translation, TMD1, NBD1, and TMD2 acquire a native-like fold as individual domains. After synthesis a simultaneous increase in protease resistance arises from assembly of TMD1, TMD2, and NBD2 onto NBD1, as well as the N-terminus that is wrapped around the TMDs in the native structure [14, 52, 53]. The assembly of domains coincides with transport of CFTR from the ER to the Golgi complex and the appearance of the CFTR complex-glycosylated Golgi form.

We conducted these experiments in HEK293T cells, since biosynthetic transport and the proteolytic patterns for wild-type CFTR (but also F508del) are the same in this cell line as in CF bronchial-epithelium CFBE cells [32] expressing CFTR constructs. In accord, the Balch lab [54] showed that maturation of over 60 variants from the CFTR2 database expressed in HEK293 correlated superbly (> 90%) with data obtained in Fischer rat thyroid cells [55] and HeLa cells [56]. Evidently, proteostasis is a very robust process whose features are well preserved between different cell lines.

Previous studies have shown that CFTR folds its domains largely co-translationally; insertion of the transmembrane helices occurs during translation [18, 19, 57] and the individual domains form protease-resistant structures with exception of NBD2 [7, 24, 58]. The structures [14, 17, 52], structural models [13, 15] and several biochemical studies have shown that the transmembrane domains interact with the nucleotide-binding domains through the intracellular loops that connect the transmembrane helices [59–62]. These interactions have been postulated to take place post-translationally [28, 60]. A current model suggests that after co-translational folding of individual domains, a TMD1-NBD1-R-TMD2 structure is produced, with NBD2 incorporation as a final step that is not required for cellular trafficking of CFTR [29]. Our kinetic assay allows a more detailed analysis of the order of events that occur, and we propose the following two-step model for folding (Fig. 11).

In stage 1, the first transmembrane domain of CFTR folds co-translationally, with the N- and C-termini packing with each other and with ICL1 [31]. NBD1 reaches a protease-resistant fold during translation as well [7, 24], and its N-terminal region then already interacts with TMD1 through ICL1 [31]. At this time, the N-terminus of TMD1 may well interact with NBD1 as suggested by a structural model [15] and be held by chaperones. TMD2 reaches a protease-resistant fold during translation, yet requires TMD1 and NBD1 to acquire native stability. In stage 2, ICL4 docks onto NBD1 and associates with ICL1;TMD1 and TMD2 assemble with NBD2 via ICL2 and ICL3, and all three acquire their native protease-resistant fold. The TMD1 N-terminus moves to its native position in the structure, wrapping around both transmembrane domains.

### Stage 1: co-translational folding of domains

The individual domains of CFTR fold to a large extent co-translationally [7]. The folding of the two transmembrane domains of CFTR begins with co-translational insertion of the transmembrane helices into the membrane [20], and in this study we show that their packing increases both co- and post-translationally. Immediately upon radiolabeling, CFTR is digested by Proteinase K into fragments that also arise upon digestion of individual TMDs: fragments T1a and T1c from TMD1, and T2b from TMD2. The larger T1c comprises almost the entire TMD1, sans N-terminus, suggesting that all of the six transmembrane helices are packing with each other, protecting the two intracellular loops. In-vivo studies using several membrane proteins in *E. coli* have shown that specific ‘packing’ interactions between the N- and C-terminal transmembrane helices occur during translation, and that these are essential in forming a stable tertiary structure [8]. This also applies to the CFTR TMDs, with the difference that the long intracellular loops emanating from the transmembrane helices start packing in the cytoplasm rather than in the lipid bilayer. Increased protection of the cytosolic N-terminus of TMD1 against digestion requires the presence of its cytosolic C-terminus [8]. In the closed cryo-EM structure [14], TMH6 is wedged between TMH1-3 and TMH4, separating TMH4 from TMH1-3; this may well explain the accessibility of Proteinase K to Ser256 in ICL2 and the formation of T1a, which in essence is a truncated T1c. When digested with more protease, the T1a fragment persisted, suggesting that TMH1-3 forms a stable trimeric helical bundle, an evolutionarily conserved folding unit found in multi-spanning membrane proteins [31, 63]. For CFTR, early formation of a stable TMD1 structure is important, as underscored by the mode of action of class-I correctors: VX-809 (Lumacaftor) and VX-661 (Tezacaftor) bind and stabilize TMD1 and thereby rescue mutant CFTR [7, 35, 37, 64]. TMD1 may well be required as template for assembly with the other domains for generation of a functional chloride channel.

Once NBD1 is translated, it folds quickly and starts co-translational domain assembly with TMD1 [7, 31]. The NBD1-specific immunoprecipitate contains the N1a fragment we had identified before [24], consisting of NBD1 lacking its N- and C-termini. Protease resistance of NBD1 notably preceded that of the other domains, showing that NBD1 folds and obtains its native protease resistance independent of the other domains and thus faster. The rapid and independent folding of NBD1 is underscored by the many studies on purified NBD1 [62, 65, 66] and highlights its importance as a docking scaffold for ICL1 and ICL4 of the transmembrane domains. The N-terminal subdomain of NBD1 (residues G404 – L436) improves TMD1 folding co-translationally, by improving packing of ICL1 with the N- and C-termini of TMD1 [31]. This early docking of ICL1 onto NBD1 is complemented with the later docking of ICL4 onto NBD1 around F508. This explains the multiple defects F508del causes: not only NBD1 misfolding but also disruption of an essential domain-assembly step, the interaction of ICL4 (in TMD2) with the F508 region in NBD1 [24, 29, 49, 67–70].

The early folding fragment T2b starts from the coupling helix of ICL3 to the C-terminal end of TMD2, with ICL4 being protected. The four helices comprising T2b, which are TMH9-12, together with TMH1-2 of TMD1, form the domain swap-structure seen in CFTR [14]. The N-terminal boundary of the smaller T2a fragment is near the end of the cytosolic extension of TMH10, just before the ICL4 coupling helix begins, indicating that ICL4 is at least partially exposed. This exposure may occur as ICL4 is not yet docked onto NBD1. Once ICL4 is docked onto NBD1 via the coupling helix, it starts to pack against ICL1 of TMD1.

The CFTR domains, especially the TMDs, must maintain a certain level of flexibility for interacting with the NBDs and opening and closing the channel, which is consistent with the linkers between all domains remaining exposed and cleavable throughout both stage 1 and stage 2 of the CFTR folding pathway.

### Stage 2: post-translational domain assembly

At later chase times, in stage 2 of CFTR folding, the appearance of fragments T1d–f shows protection of both ICL2 (decrease of T1a) and the N-terminus (T1d–f). Increasing protease resistance of the N-terminus is consistent with the N-terminus wrapping around the transmembrane helices of TMD1 and TMD2 [14]. TMD1, TMD2, and NBD2 simultaneously show conformational changes, with the protection of ICL2 and ICL3, and protease-resistance of NBD2. ICL2 in TMD1 and ICL3 in TMD2 start packing with each other and with NBD2, which results in simultaneous ICL2 and ICL3 protection. This gives rise to the T2c fragment, which consists of TMH8-12, almost the entire TMD2. TMH7 is not in T2c, whereas it is a stable transmembrane helix that acts as signal peptide for TMD2 [18, 71]. We hypothesize that it does associate tightly with TMH8-12 but is separated in SDS-PAGE by cleavage in ECL4, caused by the unstable TMH8 [72]. ICL2 and ICL3 may interact before binding to NBD2. A previous study showed that CFTR without NBD2, exposing ICL2 and ICL3 to the cytoplasm, exits the ER [29]. For CFTR to be released from chaperones, pass quality control and exit the ER, hydrophobic amino acids need to be buried inside the structure, and this can be accomplished by ICL2-ICL3 lateral packing. Even in the presence of NBD2, however, ICL2 and ICL3 remain somewhat exposed to protease after their assembly, as evidenced by some persistence of T2b and T1a, which arise from cleavage in ICL3 and ICL2, respectively. This suggests that the interfaces between NBD2, ICL2, and ICL3 remain less packed than those of ICL1, ICL4, and NBD1. The cryo-EM structures, however, suggest a larger (and hence stronger) interface at the coupling interfaces of ICL2 and ICL3 onto NBD2 than of ICL1 and ICL4 onto NBD1 [52]. This hints at the lateral packing of ICL1 with ICL4 perhaps being stronger than that of ICL2 with ICL3.

NBD2 and NBD1 are very similar in structure, and one would expect that they follow the same folding pathways. Yet, we here show that NBD2 in CFTR did not fold independently and required the other domains to fold. It acquired protease resistance through assembly with the TMDs in stage 2 of CFTR folding, and hence with slower kinetics than NBD1. This difference between the NBDs is underscored by three conspicuous features. First, NBD1 contains a regulatory insertion that is absent in NBD2 and which in F508del affects maturation of the mutant channel. RI deletion rescues F508del CFTR to the cell surface [73, 74] but effects on folding nor function of wild-type CFTR have been reported. RI enables NBD1 to adopt an alternative conformation that is in equilibrium with the canonical fold; it affects stability of the domain and likely regulates channel function [75]). Interesting is the size similarity of the proteolytic fragments deriving from the 2 NBDs, N1a and N2a, especially because N1a arises from cleavage of RI and regulatory extension RE, both lacking in NBD2. This implies that upon domain assembly, NBD1 and NBD2 share a compact core, and expose cleavable sequences in similar places, whether disordered or not.

A second difference is that isolated NBD2 is a much less stable domain than NBD1 and requires multiple solubilizing mutations to produce recombinantly for example for determination of its structure [76]. Third, compared to NBD2, NBD1 lacks a segment containing a β-strand and an α-helix, which shrinks the cleft into which ICL4 of TMD2 docks. As a consequence, the NBD1/TMD interface is weaker than the NBD2/TMD interface [52]. This larger interface with the TMDs may lend the stability NBD2 needs to fold. Assembly of NBD2 with the two TMDs may require prior assembly of TMD1 and TMD2, with each other and with NBD1. The interfaces between the transmembrane helices of TMD1 and TMD2 are complex and may require transmembrane-helix reorganization and membrane-lipid displacement, perhaps explaining why CFTR domain assembly is such a lengthy process.

### Analysis of CFTR mutants and modulator responses

Incorporation of the new TMD antibodies into the coupled pulse chase-limited proteolysis assay has allowed us to look at higher resolution into consequences of mutations in CFTR on conformations of the maturing protein. We here show that the most prevalent and well-studied disease-causing mutation F508del, which established cystic fibrosis as protein-folding disease, caused co-translational misfolding of NBD1 but not TMD1 nor TMD2. Our assay uncovered that folding of the first nucleotide-binding domain precedes that of the other domains and that folding of NBD1 is the Achilles' heel in the folding pathway of the entire polytopic multispanning membrane protein. Deletion of the single F508 residue causes the complete absence of stage-2 folding in that F508del NBD1 does *not* support assembly of TMD1, TMD2, and NBD2.

The staging of CFTR's folding pathway in two experimentally distinguishable phases possibly opens opportunities for therapeutic interventions. Salvage of a functional channel is achievable through targeting either folding of NBD1 itself or alternatively assembly of F508del NBD1 with the other domains into a (partially) active structure [49, 77]. Our data are in support of this concept, as the combination of the two corrector drugs VX-809 and VX-445 rescued F508del CFTR by enhancing domain assembly (stage 2) without restoring NBD1 folding, which included the docking of ICL1 and ICL4 onto misfolded F508del NBD1. Despite the absence of F508, the docking surface of NBD1 must have been sufficient to allow its assembly with TMD1 and TMD2, and permit TMD assembly with NBD2 like in wild-type CFTR. Whereas VX-809 acts primarily on TMD1 [17, 31, 35, 37, 64, 78], the mode of action of VX-445 is less clear. It was reported to bind NBD1 and/or TMDs and is thought to work late, at the level of domain assembly [51, 53, 79, 80]. We do not find rescue of NBD1 by VX-445 but confirmed a stimulating effect of domain assembly.

The assay also demonstrated that the DXD motif in NBD1 that was identified to be required for export of CFTR from the ER in fact was required already *upstream* of export: for proper domain folding and assembly, upstream of transport. CFTR mutated in this DAD export motif phenocopied the misfolding and degradation of F508del CFTR. We also examined the mutants of the diacidic ER export signal (DAD) that accumulate in the ER and fail to be recognized by the COPII machinery. This mutation initially was thought to encode a relatively pure sorting mutant as opposed to a conformational mutant such as F508del CFTR [44] and thus should be primarily defective at the level of Sec24 binding. More recent findings, however, show enhanced interaction of an ER-export mutant with Get4 [45], which together with Bag6 and Ubl4 promotes degradation of misfolded ER proteins [81]. Moreover, the L558S mutant close to the diacidic DAD motif within NBD1 is misfolded [50]. In accord with this central role of NBD1 in the assembly of CFTR domains, we now show that mutants of the DAD export motif fail to fold NBD1 and as a result fail to assemble their domains correctly, suggesting that misfolding prevented entry of CFTR into the COPII ER-export vesicles, rather than the mere absence of an export signal.

Collectively, we show that a repertoire of domain-specific antibodies together with a coupled pulse chase-limited proteolysis immunoprecipitation assay constitutes a powerful method to uncover folding pathways in vivo in a temporal manner, to investigate folding-function relationships of (mutant) proteins, and uncover conformational effects of modulator drugs. We have shown CFTR folding to encompass 2 discrete stages, a highly modular process of domain folding and stepwise domain assembly. On the one hand, this staging has the disadvantage that a mutation like F508del not only destroys the folding of one domain, NBD1, but also blocks entry into stage 2. On the other hand, not all domain interfaces are crucial, and the advantage is that the other 3 domains can rescue the complete protein by assembling and thereby stabilizing the defective domain. The modulator rescue of F508del CFTR domain assembly around a still defective NBD1 explains the relatively high fidelity of folding and the importance of a step-wise folding process for such complex proteins. While our paper was under revision, Fiedorczuk and Chen published the structure of F508del CFTR with bound Trikafta modulators in which they showed amongst others and consistent with our observations that the NBD1-TMD interface of pharmacologically corrected F508del CFTR is distinct from that of wild type CFTR [17].

We provide experimental evidence from intact cells for a fundamental principle in protein folding shown before by in-vitro refolding studies: that low-contact-order interactions are formed before high-contact-order folding. The pathway of domain folding before domain assembly that CFTR follows makes sense from a point of view of fidelity of folding, order of folding, and it fits with in-vitro folding principles and models such as the hydrophobic-collapse and the nucleation-condensation models of folding. Properties of specific ABC transporters such as the unstructured regulatory region, the number and position of N-linked glycans, or the presence of intramolecular disulfide bonds all may affect folding. Yet, given the high structure conservation of the features that are dominant in CFTR folding, we anticipate the 2-stage folding process to be very similar for other ABC transporters and likely for other multi-spanning membrane proteins as well.

## Materials and methods

### Antibodies and reagents

Peptides from the first extracellular loop of TMD1 (aa A107 –S118), C-terminus of TMD1 (aa S364–K381), ICL4 in TMD2 (aa Q1035–S1049), and the C-terminal part of TMD2 (aa E1172 – Q1186) were selected with the AbDesigner algorithm of NIH (https://hpcwebapps.cit.nih.gov/AbDesigner/) and synthesized as described [82]. The purity of synthetic peptides was analyzed by HPLC and mass spectrometry. Synthetic peptides were coupled to rabbit serum albumin (RSA) with the linker Sulfo-*m*-maleimidobenzoyl-*N*-hydroxysuccinimide ester (Sulfo-MBS, Thermo Scientific) in a carrier(1):linker (50):peptide (50) molar ratio. RSA and Sulfo-MBS were dissolved separately in 500 μl of PBS at pH 8.4, mixed and incubated at room temperature for 45 min. RSA-sulfo-MBS was desalted on a PD10 column (GE Healthcare) with PBS at pH 7.2, and concentrated to 1 mL (Vivaspin 20, GE Healthcare). Desalted RSA-sulfo-MBS was added to the peptides, which were dissolved in 200 μl of PBS, pH 7.2, and incubated for 2 h at room temperature [83]. The RSA-coupled peptides were used for antibody production (Pocono rabbit farm & Laboratory, Canadensis, PA). Supplementary Table 1 lists all antibodies used throughout this paper. Corrector compounds VX-809 and VX-445 (Selleck Chemicals) were dissolved in DMSO and stored at − 80 °C. Proteinase K from Tritirachium Album (quality level ELITE) was purchased from Sigma.

### Expression constructs

CFTR single-domain constructs were generated in pBS vectors as previously described [7] and subcloned into pBI-CMV2 using NotI and XhoI. The hCFTR construct in pBI-CMV2 was a kind gift of Linda Millen and Dr. Phil Thomas (University of Texas Southwestern Medical Center, USA). The TMD2-3HA construct was generated by Gibson assembly from pBI-CMV2-CFTR and a gene block consisting of triple HA, with 3xHA positioned at position S898 in ECL4 of CFTR. The TMD1-L-TMD2 construct also was generated by Gibson assembly from pBI-CMV2-1202X, pcDNA3.1-Pgp, and included aa M1–M394 (TMD1) and aa M837–D1202 (TMD2) from CFTR, and aa 626–683 from MDR1 in between that acts as a linker. N-terminally truncated constructs were generated from pBI-CMV2-CFTR by PCR. PCR products then were cloned into pBI-CMV2 using AflII and NheI. To retain the same 5’-UTR we also generated a wild-type CFTR with the same cloning strategy. C-terminally truncated constructs were generated from pBI-CMV2-CFTR by PCR and cloned into pBI using NotI and SalI. CFTR mutations were made from pBI-CMV2-CFTR template using high-throughput PCR mutagenesis [84]. In short, two fragments are created using the AmpR forward primer in combination with the mutant reverse primer and vice versa and subsequently ligated together using Gibson assembly [85]. A full list of PCR primers is described in the supplementary materials (Table S7). Construct nomenclature: N-terminal truncation ΔN48 starts with methionine followed by residues 49 and following. C-terminal truncation K381X has a stop codon instead of lysine at position 381, and therefore has 380 as the most C-terminal residue.

### Cell culture and transfection

HEK293T cells were maintained in DMEM supplemented with 10% FBS and 2 mM GlutaMAX (growth medium) and incubated at 37 °C with 5% CO_2_. Cells were seeded onto polylysine-coated 6 cm dishes to reach 70% confluency and transfected using linear 40 kDa polymer polyethylenimine (PEI) as described [25]. After 4 h, the transfection mix was replaced with growth medium and cells were cultured for 16–20 h prior to experiments.

### Radioactive pulse and chase

HEK239T cells were used in pulse-chase assays as described [86, 87]. Cells were pulse labeled for 15 min with 132 μCi/6 cm dish with EasyTag Express ^35^S Protein Labeling Mix (Perkin Elmer). Radiolabeling was stopped by adding excess, unlabeled 5 μM methionine and 5 μM cysteine. At indicated chase times, cells were washed twice with ice-cold Hanks' balanced salt solution (HBSS, Life Technologies) and solubilized in ice-cold lysis buffer (20 mM MES, 100 mM NaCl, 30 mM Tris–HCl pH 7.4, 1% Triton X-100) without protease inhibitors. Nuclei were removed by centrifugation for 10 min at 4 °C in a microfuge at maximum speed, and the supernatant was subjected to limited proteolysis or immunoprecipitation.

### In-vitro translation and translocation

Target mRNA was prepared by transcribing DNA using T7 RNA polymerase according to the manufacturer’s instructions (Promega). In-vitro translations were done with rabbit reticulocyte lysate (Flexi Rabbit Reticulocyte Lysate System, Promega) and translated CFTR (domains) were translocated and inserted into HEK293T-derived microsomes or semi-permeable HEK293T cells as source of ER membrane as described [7]. In brief, target mRNA was added to the reaction mix containing rabbit reticulocyte lysate, 10 μCi/μL EasyTag Express ^35^S Protein Labeling Mix (Perkin Elmer), and membranes. The reaction proceeded at 30 °C for 30 min, was stopped with 1 mM cycloheximide, and membranes were pelleted through centrifugation at 10,000 × g for 3 min at 4 °C. Newly translated proteins were retrieved from the pellet fraction by dissolving the membranes in 10 μL KHM (110 mM KOAc, 2 mM Mg(OAc)_2_, 20 mM HEPES pH 7.2) containing 1% Triton X-100 for 10 min at 4 °C. Samples were either subjected to immunoprecipitation or used directly for SDS-PAGE analysis after adding 2 × Laemmli sample buffer.

### Preparation of semi-permeabilized cells

The preparation of semi-permeabilized cells was described before [88]. Confluent HEK293T cells (~ 80%) from a 10-cm dish were trypsinized, resuspended in 9 mL ice-cold KHM containing 10 µg/mL Soybean Trypsin Inhibitor (Sigma) and transferred to a 15-mL polypropylene tube. Starting from this step, the cells were kept on ice and centrifugation was done at 4 °C. Cells were spun down at 250 × g for 3 min and resuspended in 6 mL ice-cold KHM. To selectively permeabilize the plasma membrane, 40 µg/mL digitonin (Calbiochem) was added and cells were mixed by inversion. After exactly 5 min incubation on ice, permeabilization was stopped by adding 8 mL ice-cold KHM. Cells were immediately spun down, resuspended in 10 mL ice-cold HEPES buffer (50 mM HEPES pH 7.2, 90 mM KOAc), and incubated for 10 min on ice. The semi-permeable cells then were resuspended in 1 mL ice-cold KHM and transferred to a 1.5-mL tube. To check whether permeabilization succeeded, Trypan Blue (Fluka) was added to the cells and the fraction of permeable cells counted by light microscopy. Next, the semi-permeable cells were briefly spun down for 15 s at 10,000 × g and resuspended in 100 µL KHM. To degrade endogenous mRNA, 1 mM CaCl_2_ and 10 µg/mL micrococcal nuclease (GE Healthcare) were added and incubated for 12 min at room temperature. To inactivate micrococcal nuclease, 4 mM EGTA was added to chelate calcium. After a final spin down and resuspension in KHM, cells were used in the in-vitro translation-translocation assay.

### Endoglycosidase H and PNGase F treatment

After in-vitro translation, the membrane fraction containing translocated CFTR was dissolved in 10 µL 100 mM NaOAc (pH 5.5) and 1% Triton X-100. 500 U of Endoglycosidase H was added and incubated at 37 °C for 1 h. Samples were used for SDS-PAGE analysis after adding 2 × Laemmli sample buffer. PNGase F treatment was performed after immunoprecipitation. The beads were resuspended in PBS containing 0.2% SDS and heated for 5 min at 55 °C. Triton was added at a final concentration of 2% to quench the SDS, and 1 U of PNGaseF was added and incubated at 37 °C for 1.5 h. Samples were used for SDS-PAGE analysis after adding 5X sample buffer.

### Limited proteolysis

Limited proteolysis was performed as described [7, 24]. In brief, lysates were treated with 25 μg/mL Proteinase K (Sigma-Aldrich) for 15 min on ice. Proteolysis was stopped by mixing equal volumes of lysis buffer supplemented with 2 mM PMSF and 2 μg/mL CLAP (chymostatin, leupeptin, antipain and pepstatin (Sigma-Aldrich)) with the lysates. Protein aggregates were pelleted by 16,000 × g centrifugation for 5 min at 4 °C, and the supernatant was used for immunoprecipitation.

### Immunoprecipitation

Antibodies against CFTR were pre-incubated with protein-A or protein-G Sepharose beads (GE Healthcare) for 15 min at 4 °C before adding protease-treated or non-treated lysates. The lysates were added to the antibody-beads mixtures and incubated at 4 °C for either 3 h or overnight. The beads were washed twice for 15 min at room temperature. A list of immunoprecipitation conditions is provided in the supplementary materials (Table S8). Beads were resuspended in 10 μl 10 mM Tris–HCl pH 6.8 containing 1 mM EDTA, and immune complexes were eluted by adding 10 μl 2 × reducing Laemmli sample buffer (final concentration: 200 mM Tris–HCl pH 6.8, 3% SDS, 10% glycerol, 1 mM EDTA, 0.004% bromophenol blue, and 25 mM DTT) and heating for 5 min at 55 °C.

### SDS-PAGE and autoradiography

Samples generated by in-vitro translation or fragments generated by limited proteolysis were resolved by 12% SDS-PAGE, whereas all other samples were resolved by 7.5 or 10% SDS-PAGE, as indicated in the legends. Gels were dried and exposed to super-resolution phosphor screens (Fuji Film) for quantifications, or to Kodak MR films for manuscript figure images. Signals from screens were visualized with a Typhoon FLA-7000 scanner (GE Healthcare Life Science) and quantified with ImageQuantTL software (GE Healthcare Life Science).

### Structural analysis

The helical propensity of domain sequences was determined using secondary structure prediction algorithms via JPred v4 [89]. Information of secondary structures of NBD1 and NBD2 of CFTR were taken from PDB (5UAK). Images of protein structures were created using UCSF Chimera [90].

### Supplementary Information

Below is the link to the electronic supplementary material.

Supplementary file1 (PDF 4393 KB)

## Data Availability

The datasets generated during and/or analyzed throughout the paper are not publicly available due to the complexity of the data but are available from the corresponding author on reasonable request.
