# Supplementary material for: ABC-transporter CFTR folds with high fidelity through a modular, stepwise pathway
Source: Cell Mol Life Sci. 2023 Jan 7;80(1):33. doi: 10.1007/s00018-022-04671-x (PMC9825563; doi:10.1007/s00018-022-04671-x)
Supplement: Supplementary file 1 — Supplementary file1 (PDF 4393 KB) [file 18_2022_4671_MOESM1_ESM.pdf]

SUPPLEMENTARY TEXT & TABLES

Workflow for identification of domain-specific protease-resistant fragments

To identify the regions in CFTR that are protected or de-protected from the protease during folding we determined the identity of each proteolytic fragment, i.e. the cleavage sites used to generate the domain-specific protease-resistant fragments. We mapped the presence of antigenic epitopes and glycans in the fragments, electrophoretic mobility shifts in the fragments in missense-mutated CFTR and truncated CFTR constructs, and considered helical propensity and consensus cleavage sites of the protease to locate the cleavage sites even further. With this information we identified each cleavage site, down to just a few amino acids for most fragments, a treasure trove for many future studies.

We set out to determine the identity of each proteolytic fragment and started by mapping the presence of antigenic epitopes (Table S1, Figures 3a, 5a, 7c) and glycans in each fragment. In a next step we truncated CFTR domains or parts and analyzed resulting electrophoretic mobility shifts in SDS-PAGE, which allowed determination of several fragment boundaries already (Tables 1, S3-6 and Figures 3, 5e, 7d). Last experimental dataset was the mapping of fragment mobility shifts (or lack thereof) in point mutants and in truncated CFTR (Tables 1, S3-6 and Figures 3d, 5, 8b). These results, combined with literature on Proteinase-K consensus cleavage sites (Table S2) and alpha-helical-propensity predictions, allowed determination of the amino-acid sequences (up to a single likely residue) that constituted the N-terminal and C-terminal cleavage sites for each fragment (Tables 1, S3-6 and Figures 4a, 6a and 7a-b).

We found that the electrophoretic mobility of fragments (especially from TMD) was not dictated by size/mass alone, but strongly by charge and hydrophobicity as well. For instance, the electrophoretic mobility of fragment T1a suggested a size of ~19 kDa, but this was too small to fit within the experimentally determined fragment boundaries (minimal 23 kDa). We therefore did not use this as a strict parameter to determine cleavage sites.

Our analysis depends fully on the specificity of Proteinase K. Other proteases such as trypsin, chymotrypsin, endoGlu-C, and thermolysin are useful to confirm our findings, but the strength of Proteinase K is its low specificity, with possible cleavage sites all throughout the CFTR sequence. This implies that protection from cleavage can be caused only by conformational or external factors. Proteases select against helical sequences [1, 2], which we used by predicting helical propensity of cleavage stretches using Jpred (http://www.compbio.dundee.ac.uk/jpred/). Cleavage sites are shielded by interaction with polypeptide chains, either within the molecule (domain folding and domain assembly), or from another molecule, for instance by assembly with other subunits in an oligomer or by a (transient) interaction with another protein such as a chaperone. Our analysis in Triton X-100 favors intramolecular interactions and tightly associating oligomers and disfavors chaperone associations. We therefore conclude that proteolytic changes are caused by CFTR folding, although we cannot exclude intermolecular interactions.

The consensus cleavage sites of Proteinase K have been well established but unfortunately some ambiguity remains. Proteinase K preferentially cleaves at the carboxyl side of residues A, Q, M, C, W, S, F or L (Table S2) [3-7] located in accessible loops and not in secondary structure, especially α- 221 helices [1, 2]. Cleavage of residues that are followed by small amino acids such as alanine or glycine is slightly preferred whereas cleavage is inhibited when the residue is followed by a proline and slightly reduced when followed by valine [8]. Also, cleavage is more efficient when the cleavage site is flanked by at least 1 to 3 residues N- or C-terminal [4, 8].

The antigenic epitopes and truncated constructs provided the primary information on the preferred cleavage sites and are written in Tables 1, S3-6 (and Figures 4a, 6a and 7a-b). Next are the consensus Proteinase-K sites, and the electrophoretic mobility shifts caused by missense mutations. The reasoning for the N- and C-terminal boundaries of each fragment is in Tables S3-6 and shown in Figures 4a, 6a and 7a-b.

A missense mutation can influence proteolysis by three mechanisms:

1. Removal or addition of a consensus Proteinase-K cleavage site,

2. Change of electrophoretic mobility by charge or changed SDS binding, and
3. Change of conformation or interactions with other proteins, leading to different shielding of consensus sites.

Ad 1. Change of a cleavage site may go unnoticed when a cluster of sites is being used by the protease, implying that we cannot conclude that a lack of change equates a lack of cleavage of the target or mutated residue. Exceptions to this are the few instances where a single residue appears to be the protease target rather than an amino-acid cluster. A change in proteolytic fragment caused by a mutated cleavage site does provide positive information.

Ad 2. Electrophoretic mobility of proteins in SDS-PAGE [9] in principle is determined by their mass because of their inherent capacity to bind 1.4 g of SDS per g polypeptide, independent of amino acid composition. As SDS adds 2 negative charges it in principle overrules any charge the protein may have. These ideal conditions often are not met, for instance in hydrophobic proteins, where SDS binding may be higher or lower, or in negatively charged proteins such as all calcium-binding chaperones in the endoplasmic reticulum, where SDS binding is lower, leading to decreased mobility and overestimation of their masses [10, 11]. Analyzing smaller constructs, the individual CFTR domains and proteolytic fragments, we noticed that a charge change in the fragment did contribute to a mobility change, either up or down, by a combination of its own charge and the changed SDS-binding. On top of the charge changes we have noticed electrophoretic mobility changes unrelated to charge, such as in I539T [12]. In summary, this parameter, while important to notice and include in the analysis, does not provide rigorous information on proteolytic fragments. Mobility appeared most useful to establish whether the mutated residues were present in a fragment.

Ad 3. Limited proteolysis classically has been used for this parameter: to determine folded state or domain boundaries of proteins [1]. In Cleveland analysis [13] it is used as unique protein identifier, on the premise that each native protein has its own sequence and hence unique proteolytic pattern. We have used the approach and found a surprising capacity to zoom into the used cleavage sites. This is fortunate as mass spectrometry so far has had limited success in determining identities of these subpicomole quantities of radiolabeled fragments. Equating proteolysis to folded

state of CFTR is only possible when other proteins are not interacting and shielding protease sites. As Triton X-100 dissociates most chaperone-client and many other complexes, we limited our conclusions here to intramolecular shielding, i.e. folding.

Following this list of parameters for the N-terminus and C-terminus of each proteolytic fragment, described in Tables S3-6 and visualized in Figures 4a, 6a, 7a and 8a, we identified each fragment with rather precise boundaries (Table 1).

Figure S1

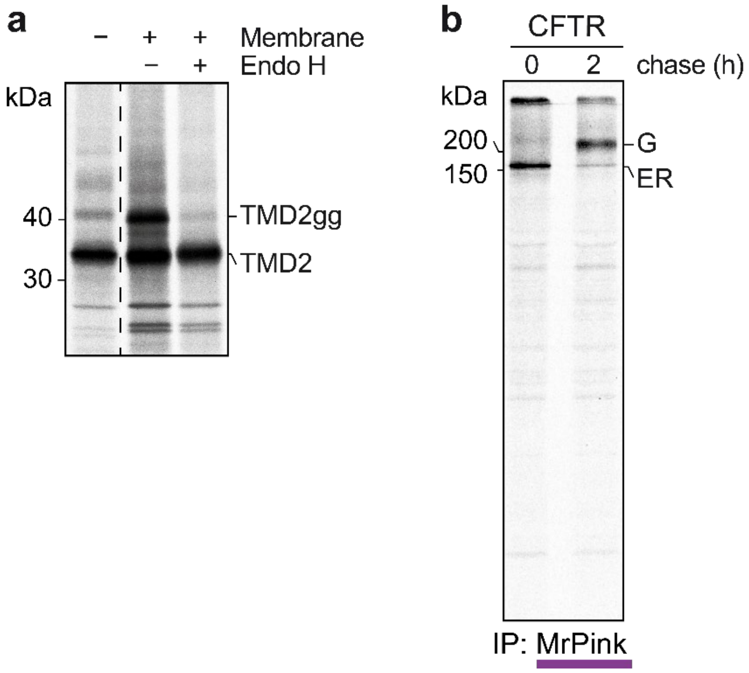

Figure S1. Glycosylation of TMD2 in vitro and CFTR in cells, related to Figure 1. **(a)** TMD2 was translated in vitro in the presence of HEK293 microsomes. Membranes were pelleted, digested with Endo H where indicated, and resolved by 12% SDS-PAGE. TMD2gg: core-glycosylated TMD2. **(b)** HEK293T cells expressing CFTR were pulse-labeled for 15 minutes and lysed immediately or after a chase of 2 hours, immunoprecipitated using MrPink antibody and resolved by 10% SDS-PAGE.

Figure S2

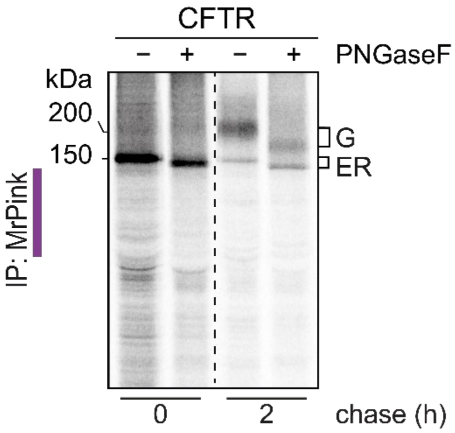

Figure S2. PNGase F deglycosylates core and complex glycosylated CFTR, related to Figure 5.

HEK293T cells expressing full-length CFTR were pulse-labeled for 15 minutes and lysed immediately or after a chase of 2 hours. CFTR was immunoprecipitated with MrPink, subjected to PNGase F treatment, and resolved by 7.5% SDS-PAGE.

Figure S3

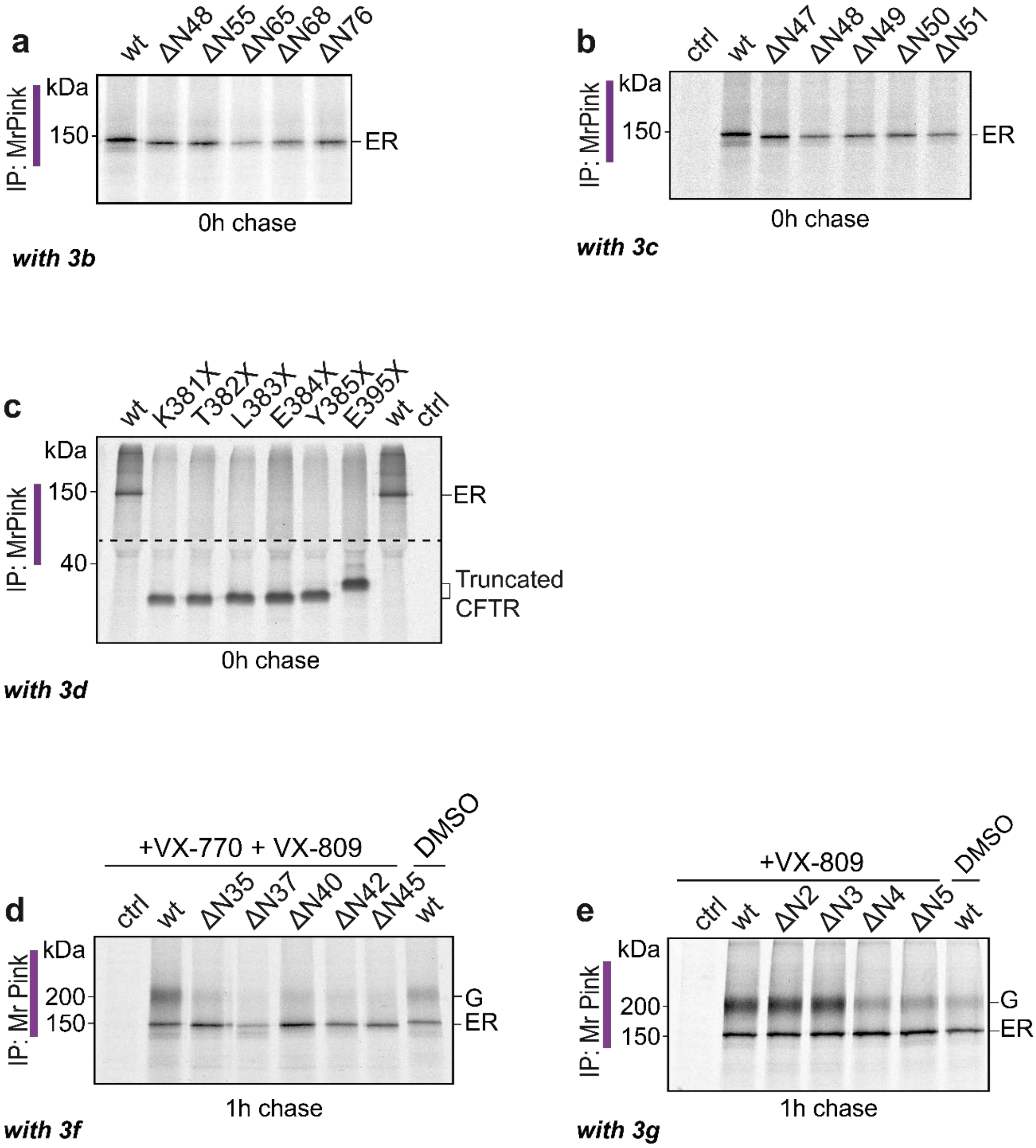

Figure S3. Protease resistant fragments shift downwards in N-terminally truncated CFTR, related to Figure 3d-g.

(a) HEK293T cells expressing N-terminal CFTR truncations ΔN48 to ΔN76 were pulse-labeled for 15 minutes and lysed immediately. CFTR variants were immunoprecipitated with MrPink and analyzed by 7.5% SDS-PAGE. Digests are shown in Figure **3b**. **(b)** Same as **(a)** but now with N-terminal CFTR truncations ΔN47 to ΔN51. Digests are shown in Figure **3c**. **(c)** same as **(a)** but with C-terminal truncations K381X to E395X. Digests are shown in Figure **3d**. **(d)** same as **(a)** but for

N-terminal truncations ΔN35 to ΔN45, and after a chase of 1 hour. Digests are shown in Figure **3f**. **(e)** same as **(d)** but now for N-terminal truncations ΔN2 to ΔN5. Digests are shown in Figure **3g**. Experiments in panels **(d)** and **(e)** were done in the presence of VX-770 (3 μM) and/or VX-809 (3 μM) as indicated.

Figure S4

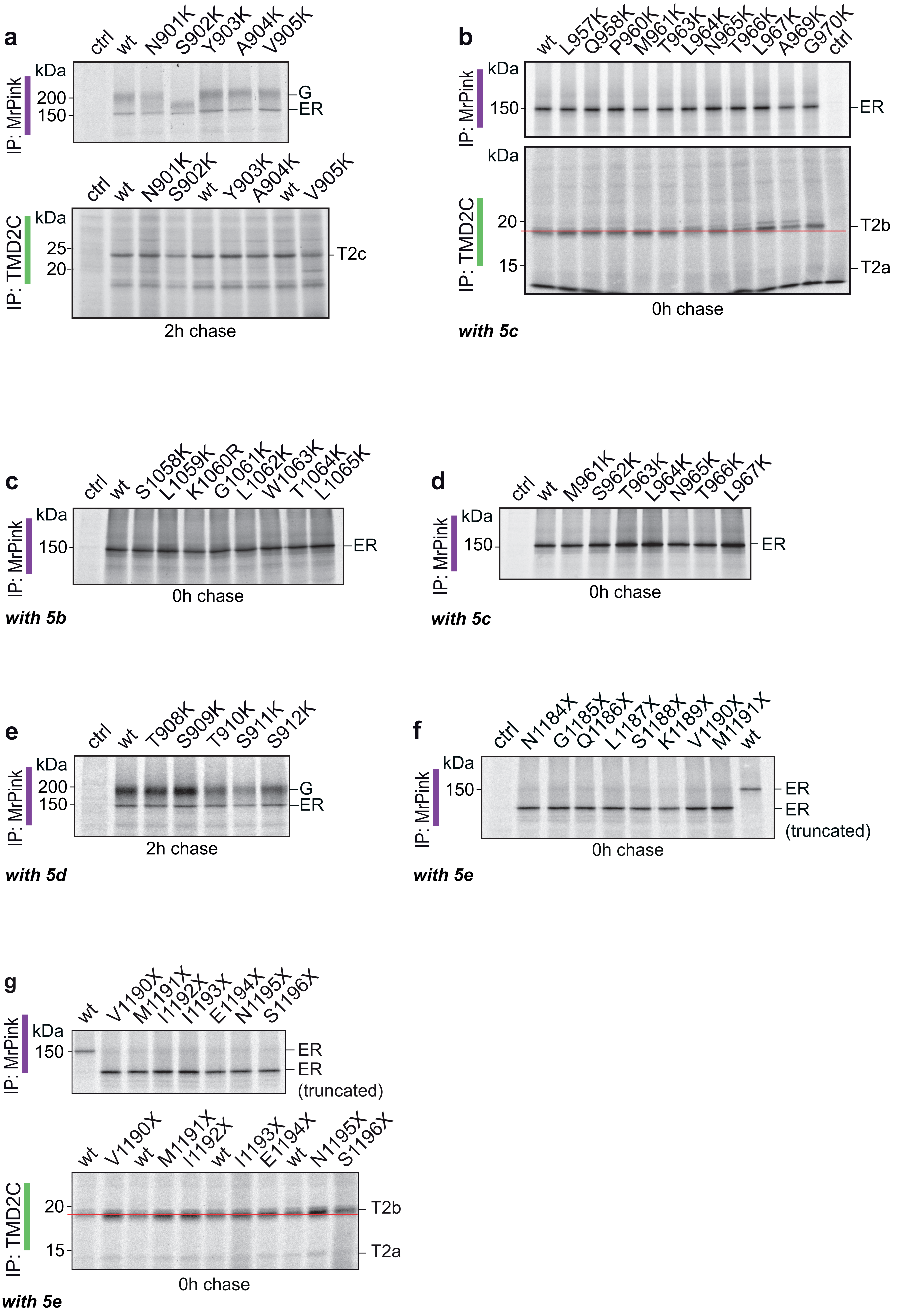
a
ctrl wt N901K S902K Y903K A904K V905K
IP: MrPink
kDa
200
150
G
ER
ctrl wt N901K S902K wt Y903K A904K wt V905K
IP: TMD2C
kDa
25
20
T2c
2h chase
b
wt L957K Q958K P960K M961K T963K L964K N965K T966K L967K A969K G970K ctrl
IP: MrPink
kDa
150
ER
IP: TMD2C
kDa
20
15
T2b
T2a
0h chase
with 5c
c
ctrl wt S1058K L1059K K1060R G1061K L1062K W1063K T1064K L1065K
IP: MrPink
kDa
150
ER
0h chase
with 5b
d
ctrl wt M961K S962K T963K L964K N965K T966K L967K
IP: MrPink
kDa
150
ER
0h chase
with 5c
e
ctrl wt T908K S909K T910K S911K S912K
IP: MrPink
kDa
200
150
G
ER
2h chase
with 5d
f
ctrl N1184X G1185X Q1186X L1187X S1188X K1189X V1190X M1191X wt
IP: MrPink
kDa
150
ER
ER (truncated)
0h chase
with 5e
g
wt V1190X M1191X I1192X I1193X E1194X N1195X S1196X
IP: MrPink
kDa
150
ER
ER (truncated)
wt V1190X wt M1191X I1192X wt I1193X E1194X wt N1195X S1196X
IP: TMD2C
20
15
T2b
T2a
0h chase
with 5e

Figure S4. Protease resistant fragments shift in CFTR mutants, related to Figure 5b-e.

(a) HEK293T cells expressing CFTR mutants N901K-V905K were pulse labeled for 15 minutes and lysed after a 2-hour chase. Detergent lysates were immunoprecipitated with MrPink or subjected to limited proteolysis with 25 µg/mL P roteinase K and immunoprecipitated with TMD2C. Top panel represents non-digested material resolved by 7.5% SDS-PAGE and the bottom panel shows the fragments generated by limited proteolysis and resolved by 12% SDS-PAGE. **(b)** same as **(a)** but with mutants L957K to G970K and 0-hour chase. Dotted red line through T2b fragment is for visual aid. **(c)** HEK293T cells expressing CFTR mutants S1058K to L1065K were pulse labeled for 15 minutes and lysed immediately. Detergent lysates were immunoprecipitated with MrPink and resolved by 7.5% SDS-PAGE. **(d)** same as **(c)** but with mutants M961K to L967K. **(e)** same as **(c)** but with mutants T908K to S912K and after a chase of 2h. **(f)** same as **(bc)** with C-terminal truncations N1184X to M1191X. **(g)** same as **(b)** but with mutants V1190X to S1196X. Dotted red line through T2b fragment is for visual aid. Digests of panels **c-f** are shown in Figure **5b-e**.

SUPPLEMENTARY TABLES

Table S1. CFTR antibodies and antigenic epitopes

**Antibody**	**Domain**	**Epitope** (residues)	**Source**
MM13-4	TMD1, N-terminus	G27-L34 [14]	Millipore
E1-22	TMD1, ECL1	A107-S118	This paper
TMD1C	TMD1, C-terminus	S364-K381	This paper
3G11	NBD1, N-subdomain	N396-F405 [15]	W.E. Balch
7D12	NBD1, α- helical subdomain	S531-V540	P. Thomas
MrPink	NBD1, whole	Multiple in NBD1 [12]	Generated in Braakman-Van der Sluijs lab
G449	R region & NBD1 C-subdomain	F653-K716 [16]	A. Nairn
570	R region	P731-S742 [14]	J. Riordan & CFF
217	R region	I807-E819 [14]	J. Riordan & CFF
I4N	TMD2, ICL4	Q1035-S1049	This paper
TMD2C	TMD2, C-terminus	E1172-Q1186	This paper
596	NBD2, N-subdomain	W1204-T1211 [14]	J. Riordan & CFF
2-39.14	NBD2, C-subdomain	E1371-R1385 (E. Sorscher)	E. Sorscher
2-3.5	NBD2, C-subdomain	V1379-T1387 (E. Sorscher)	E. Sorscher

Numbers between brackets refer to supplementary references

Table S2. Proteinase-K consensus cleavage studies

Summary of literature describing which amino acids (displayed as one-letter code) Proteinase K preferentially cleaves. Amino acids that were not or barely cleaved are in regular font. This was generally consistent throughout all studies. We chose to use the residues in bold as preferentially cleaved by Proteinase K, mostly based on the study performed by Whittaker et al., which is the most rigorous and complete one. These are colored blue in Figures 4 and 6. Results on tyrosine (Y) were too variable to consider Y a consensus cleavage site.

Study	Preferential cleavage after amino acids
[7]	**A>Q>M>C>W>S>F>L**>H>N>E=V>T>Y> K=G>R>D>I>P
[4]	**W**>Y>**F>L>A**> K>R>V>G and K>F>A
[3]	Y>**A=L**> R
[5]	Y>**F>L**> P=K=R=G
[6]	**Q-C-F-L**-Y-N-H-S-E-G> V=A=R=T=P=K

Table S3. Cleavage site identification of TMD1

Indirect, supporting results are in *italics*, conclusion is in **bold**

Fragment	N- or C-terminus	Results	Reference
T1a	N	• Does not contain MM13-4 epitope (aa 27-34).<br>• Shifts on SDS-PA gel in N-terminal truncations: ∆N48 (no shift), ∆N55 (shift down).<br>• Shifts on SDS-PA gel in N-terminal truncations: ∆N47 (no shift), ∆N48 (no shift), ∆N49 (no shift), ∆N50 (shift down), ∆N51 (shift down).<br>• *Lh2 (aa46-63) has relatively low helical propensity.*<br>• *Proteinase-K cleavage sites between aa46-55 are A46, L49, S50 and L53.*<br>• **N-terminal cleavage site is L49.**	Fig. 3a<br>Fig. 3b<br>Fig. 5b in [17]<br>[18]<br>Table S2
	C	• Contains ECL1 epitope (aa107-118), does not contain TMD1C epitope (aa362-381).<br>• Shifts on SDS-PA gel in C-terminal truncations: 249X (shift down), 307X (no shift).<br>• No shift in C-terminal truncations (357X, 366X, 376X, 386X, 395X) .<br>• Shifts on SDS-PA gel in C-terminal truncations: 254X (shift down), 255X (shift down), 256X (no shift), 257X (no shift), 258X (shift up), 259X (no shift), 260X (no shift), 261X (no shift).<br>• *ICL2 has a high helical propensity, apart from aa275-278: which is the loop after the ICL2 coupling helix.*<br>• **C-terminal cleavage site is S256.**	Fig. 3a<br>Fig. 4b in [17]<br>Fig. 7b in [17]<br>Fig. 5c in [17]<br>[18]
T1c	N	• Does not contain MM13-4 epitope (aa27-34).<br>• Shifts on SDS-PA gel in ∆N55 (shift down).<br>• Shifts in N-terminus truncations: ∆N47 (no shift), ∆N48 (no shift), ∆N49 (no shift), ∆N50 (down), ∆N51 (down).<br>• *Lh2 (aa46-63) relatively low helical propensity.*<br>• *A46, L49, S50 and L53 are only Proteinase-K consensus cleavage sites between aa46-55.*<br>• **N-terminal cleavage site is L49.**	Fig. 3a<br>Fig. 3b<br>Fig. 3c<br>[18]<br>Table S2
	C	• Contains TMD1C epitope (aa362-381) does not contain 3G11 epitope (aa396-405).<br>• Shift changes in C-terminal truncations: 381X (shift down), 382X (shift down), 383X (shift down), 384X (no shift), 385X (no shift) and 395X (no shift).	Fig. 3a<br>Fig. 3f

Fragment	N- or C-terminus	Results	Reference
		• *aa 381-396 forms a flexible loop.*<br>• *Proteinase-K consensus sites between aa381-396 are the following: L383, L387 and M394. M394 is located in a β-sheet so less likely to be cleaved.*<br>• **C-terminal cleavage site is L383.**	[19]<br>Table S2
T1d	N	• No MM13-4 epitope (aa 27-34).<br>• Shift changes in N-terminal truncations: ∆N35 (no shift), ∆N45 (shift down).<br>• Shifts in N-terminal truncations: ∆N35 (no shift), ∆N37 (shift down), ∆N40 (shift down), ∆N42 (shift down), ∆N44 (shift down) ∆N45 (shift down).<br>• *aa36-45 form a flexible linker.*<br>• *S36, Q39 and S45 are only Proteinase-K consensus sites between aa30-45 (S42 is followed by V43 and therefore less likely cleaved).*<br>• **N-terminal cleavage site is S35.**	Fig. 3a<br>Fig. 3e<br>Fig. 3f<br>[19]<br>Table S2
	C	• Contains TMD1C epitope (aa362-381) does not contain 3G11 epitope (aa396-405).<br>• *aa 381-396 forms a flexible loop.*<br>• *Proteinase-K consensus sites between aa381-396 are the following: L383, L387 and M394. M394 is located in a β-sheet so less likely to be cleaved. L383 is the cleaved residue in the more open, unfolded, protease-sensitive CFTR yielding T1c.*<br>• **C-terminal cleavage site is most likely L383.**	Fig. 3a<br>[19]<br>Table S2
T1e	N	• Contains MM13-4 epitope (aa 27-34).<br>• Shift changes in N-terminal truncations: ∆N16 (present), ∆N18 (present), ∆N20 (vague), ∆N35 (down).<br>• *Proteinase-K consensus sites between aa17-27 are F17, S18, W19 and L24. F17 most likely cleaved.*<br>• **N-terminal cleavage site is most likely F16.**	Fig. 3a<br>Fig. 3e<br>Table S2
	C	• Identical to T1d.<br>• **C-terminal cleavage site is most likely L383.**	
T1f	N	• Contains MM13-4 epitope (aa27-34).<br>• Shift changes in N-terminal truncations: ∆N4 (down)<br>• Shifts in N-terminal truncations: ∆N2 (down), ∆N3 (down), ∆N4 (down), ∆N5 (down).<br>• **No N-terminal cleavage site, starts with M1.**	Fig. 3a<br>Fig. 3e<br>Fig. 3g
	C	• Identical to T1d.<br>• **C-terminal cleavage site is most likely L383.**	

Table S4. Cleavage site identification of TMD2

Indirect results are in *italics*, conclusion is in **bold**

Fragment	N- or C-terminus	Results	Reference
T2a	N	• Glycans not present.<br>• Contains TMD2C epitope (aa1172-1186), does not contain ICL4 epitope (aa1035-1049).<br>• *Shifts in ICL4 mutations on SDS-PA gel: S1058K (no shift), L1059K (absent, smaller doublet), K1060R (no shift), G1061K (shift up), L1062K (shift up, smaller doublet), W1063K (shift up, smaller doublet), T1064K (shift up) and L1065K (no shift).*<br>• *Helical propensity is low on both sides of the ICL4 coupling helix.*<br>• *Proteinase-K consensus sites in ICL4 region are the following: L1059, L1062, W1063, L1065, A1067, F1068. All residues here are relatively high on preferential cleavage of Proteinase-K.*<br>• **N-terminal cleavage site is L1059.**	Fig. 5a<br>Fig. 2e,f, 5a<br>Fig. 5b [10,11]<br>[18]<br>Table S2
	C	• Contains TMD2C epitope (aa1172-1186), does not contain 596 epitope (aa1204-1211).<br>• Shifts in C-terminal truncations on SDS-PA gel: M1191X (no shift), V1190X (not detectable), K1189X (shift down), S1188X (shift down), L1187X (shift down), Q1186X (shift down), G1185X (shift down) and N1184X (shift down).<br>• *Proteinase-K consensus sites between aa1186-1204 are L1187, S1188, M1191 and W1204. W1204 is likely blocked by P1205.*<br>• **C-terminal cleavage site is M1191.**	Fig. 5a<br>Fig. 5e<br>Table S2
T2b	N	• Glycans not present.<br>• Contains TMD2C epitope (aa1172-1186) and contains ICL4 epitope (aa1035-1049).<br>• *Helical propensity higher in N-terminal than C-terminal of ICL3 coupling helix.*<br>• *Shift changes in ICL3 mutants on SDS-PA gel shown in C-terminal half of ICL3: M961K (no shift), S962K (no shift), T963K (no shift), L964K (shift up), N965K (no shift), T966K (doublet), L967K (doublet), A969K (doublet).*<br>• *Proteinase-K consensus sites in C-terminal region of ICL3 are the following: L964, L967, A969.*<br>• **N-terminal cleavage site is L964.**	Fig. 5a<br>Fig. 2e, 2f, 5a<br>[18]<br>Fig. 5c, S4b [10,11]<br>Table S2

Fragment	N- or C-terminus	Results	Reference
	C	• Contains TMD2C epitope (aa1172-1186), does not contain 596 epitope (aa1204-1211).	Fig. 5a
		• Shift changes in C-terminal truncations:<br>S1196X (no shift), N1195X (no shift), E1194X (no shift), I1193X (no shift), I1192X (no shift), M1191X (no shift), V1190X (no shift),K1189X (shift down), S1188X (shift down), L1187X (shift down), Q1186X (shift down), G1185X (shift down) and N1184X (shift down).	Fig. 5e, S4g
		• *Proteinase-K consensus sites between aa1186-1204 are L1187, S1188, M1191 and W1204. W1204 is likely blocked by P1205.*	Table S2
		• **C-terminal cleavage site is M1191.**	
T2c		• Glycans not present.	Fig. 5a
		• Contains TMD2C epitope (aa1172-1186) and contains ICL4 epitope (aa1035-1049).	Fig. 2e, 2f, 5a
		• T2c > T2b	
	N	• *Shift changes in ECL4 charge mutants:*<br>*N901K (no shift), S902K (no shift), Y903K (no shift), V905K (no shift), T910K (no shift), S911K (shift up), S912K (shift up).*	Fig. 5d, S4a [10,11]
		• *Proteinase-K consensus sites in ECL4 region are the following: S902, A904, S909, S911, S912.*	Table S2
		• **N-terminal cleavage site is S909.**	
	C	• Identical to T2a and T2b.<br>• **C-terminal cleavage site is most likely M1191.**	

Table S5. Cleavage site identification of NBD1

Indirect results are in *italics*, conclusion is in **bold**

Fragment	N- or C-terminus	Results	Reference
N1a	N	• 25-kDa fragment also arises from digestion of in-vitro translated NBD1 (aa389-673).<br>• Contains 7D12 epitope (aa531-540), does not contain 3G11 epitope (aa396-405), so cleavage site is downstream of F405.<br>• *Disordered region RI (aa405-436) offers >10 Proteinase-K cleavage sites at its periphery, most at its C-terminus; all are likely target because of the lack of structure.*<br>• *25 kDa would translate to ~225 amino acids; if C-terminus is F653, then N-terminus could be ~L428, suggesting cleavage at S427, which is in a patch of cleavage sites: S427-LFF-S431.*<br>• **N-terminal cleavage site is in RI, between F405 and L436, with S427 likely candidate.***	Fig. 7b and [12]<br>Fig. 7b,c
	C	• 25-kDa fragment also arises from digestion of in-vitro translated NBD1 (aa389-673)<br>• Contains 7D12 epitope (aa531-540), does not contain G449 epitope (aa653-719), so cleavage site is upstream of aa653.<br>• *25 kDa would translate to ~225 amino acids, ~80% of NBD1.*<br>• *Region RE (aa638-673) adopts 2 alpha-helices in the NBD1 structure but is unresolved in full-length CFTR, which suggests it would allow cleavage.*<br>• *RE offers 15 Proteinase-K cleavage sites, spread throughout the sequence, with F653 in a series of 3 cleavage sites FSA.*<br>• **C-terminal cleavage site is in RE, likely F653.***	Fig. 7b and [12]<br>Fig. 7b,c<br>[20]

* N1a arises from cleavages in RI and RE. We cannot exclude that this fragment is shifted towards the N-terminus, with N- and C-terminal cleavage sites moved upstream. This is not relevant for the analysis however.

Table S6. Cleavage site identification of NBD2

Indirect results are in *italics*, conclusion is in **bold**

Fragment	N- or C-terminus	Results	Reference
N2a	N	• Contains 596 epitope (aa1204-1211), does not contain TMD2C epitope (aa1172-1186).	Fig. 8b
		• *M1191 is cleavage site for TMD2 fragments. Only other Proteinase-K consensus sites between aa1186-1204 are L1187, S1188, M1191 and W1204. W1204 is likely blocked by P1205. L1187, S1188 are alternatives.*	Table S2
		• **N-terminal cleavage site is M1191.**	
	C	• Contains 2-39.14 epitope (aa1370-1385) and 2-3.5 epitope (aa1379-1387).	Fig. 8b
		• Shift changes in C-terminal truncations: F1437X (shift down), R1438X (shift down), Q1439X (shift down), A1440X (shift down), I1141X (no shift), S1442X (no shift).	Fig. 8c
		• *Size of the fragment and similarity to NBD1 sµggests N2a includes almost all of NBD2 lacking the flexible C-terminus (aa1436-C term).*	
		• **C-terminal cleavage site is Q1439.**	

Table S7. Primers used for generation of constructs used in this study

Construct Name	Primer Sequence (5' to 3')
CFTR-TMD1 (CFTR 395X)	F: GGAATGCAGATGAGAATAGC<br>R: CCCTCGAGTCTACATCACTACTTCTGTAGTCG
CFTR-TMD2 (837-1202)	F: GCTCTAGAATGGAGAGCATACCAGCAGTGAC<br>R: CCCTCGAGCTAATCTTTCTTCACGTGTGAATTCTC
TMD2-3HA	F: AGAAATAACAGCTATGCAGTG<br>R: ACTATGAGTACTATTCCC
	Gene block:<br>GGGAATAGTACTCATAGTGGTACCTACCCTTACGATGTTCCTGACTAT<br>GCGGGCTATCCGTACGACGTCCCGGACTATGCAGGATCCTATCCATAT<br>GATGTGCCAGATTACGCTACTAGTAGAAATAACAGCTATGCAGTG
TMD1-L-TMD2	F: AGAATTCACACGTGAAGAAAGATTGACGGCCGCATCGATAAG<br>R: CCTGCTGTCTGCATTGTGACCATCACTACTTCTGTAGTCGTTAAGTT
	F: CGACTACAGAAGTAGTGATGGTCACAATGCAGACAGCAGG<br>R: AGGTATGTGTTCCATGTAGTAAGCTTTCTGTCTTGGGCTT
	F: GCTTATCGATGCGGCCGTCAATCTTTCTTCACGTGTGAATTCT<br>R: AAGCCCAAGACAGAAAGCTTACTACATGGAACACATACCTT
Wild-type (- 5'-UTR)	F: ATAGCTAGCACCATGCAGAGGTCGCCTCTGGAAAAG<br>R: GGCTGAGCTATTGAAGTATCTCACATAGGCTG
ΔN2	F: ATAGCTAGCACCATGAGGTCGCCTCTGGAAAAGG<br>R: CCGCTCGAGCTAATATTCCAATGTCTTATA
ΔN3	F: ATAGCTAGCACCATGTCGCCTCTGGAAAAGGCC<br>R: CCGCTCGAGCTAATATTCCAATGTCTTATA
ΔN4	F: ATAGCTAGCACCATGCCTCTGGAAAAGGCCAGCGTTGTC<br>R: GGCTGAGCTATTGAAGTATCTCACATAGGCTG
ΔN5	F: ATAGCTAGCACCATGCTGGAAAAGGCCAGCGTTGTC<br>R: GGCTGAGCTATTGAAGTATCTCACATAGGCTG
ΔN8	F: ATAGCTAGCACCATGGCCAGCGTTGTCTCCAAAC<br>R: GGCTGAGCTATTGAAGTATCTCACATAGGCTG
ΔN10	F: ATAGCTAGCACCATGGTTGTCTCCAAACTTTTTTTCAGCTG<br>R: GGCTGAGCTATTGAAGTATCTCACATAGGCTG
ΔN12	F: ATAGCTAGCACCATGTCCAAACTTTTTTTCAGCTGGACCAG<br>R: GGCTGAGCTATTGAAGTATCTCACATAGGCTG
ΔN16	F: ATAGCTAGCACCATGTTCAGCTGGACCAGACCAATTTTG<br>R: GGCTGAGCTATTGAAGTATCTCACATAGGCTG
ΔN18	F: ATAGCTAGCACCATGTGGACCAGACCAATTTTGAGG<br>R: GGCTGAGCTATTGAAGTATCTCACATAGGCTG
ΔN20	F: ATAGCTAGCACCATGAGACCAATTTTGAGGAAAGGATAC<br>R: GGCTGAGCTATTGAAGTATCTCACATAGGCTG
ΔN35	F: ATAGCTAGCACCATGGACATATACCAAATCCCTTCTGTT<br>R: GGCTGAGCTATTGAAGTATCTCACATAGGCTG
ΔN37	F: ATAGCTAGCACCATGTACCAAATCCCTTCTGTTGATTCTG<br>R: CCGCTCGAGCTAATATTCCAATGTCTTATA
ΔN40	F: ATAGCTAGCACCATGCCTTCTGTTGATTCTGCTGACA<br>R: CCGCTCGAGCTAATATTCCAATGTCTTATA
ΔN42	F: ATAGCTAGCACCATGGTTGATTCTGCTGACAATCTATCTGAA<br>R: CCGCTCGAGCTAATATTCCAATGTCTTATA
ΔN45	F: ATAGCTAGCACCATGGCTGACAATCTATCTGAAAAATTGG<br>R: GGCTGAGCTATTGAAGTATCTCACATAGGCTG
ΔN47	F: ATAGCTAGCACCATGAATCTATCTGAAAAATTGGAAAGAGAATGGG

	R: GGCTGAGCTATTGAAGTATCTCACATAGGCTG
ΔN48	F: ATAGCTAGCACCATGCTATCTGAAAAATTGGAAAGAGAATG<br>R: GGCTGAGCTATTGAAGTATCTCACATAGGCTG
ΔN49	F: ATAGCTAGCACCATGTCTGAAAAATTGGAAAGAGAATGGGAT<br>R: GGCTGAGCTATTGAAGTATCTCACATAGGCTG
ΔN50	F: ATAGCTAGCACCATGGAAAAATTGGAAAGAGAATGGGATAGAGAG<br>R: GGCTGAGCTATTGAAGTATCTCACATAGGCTG
ΔN51	F: ATAGCTAGCACCATGAAATTGGAAAGAGAATGGGATAGAGAG<br>R: GGCTGAGCTATTGAAGTATCTCACATAGGCTG
ΔN55	F: ATAGCTAGCACCATGGAATGGGATAGAGAGCTGGCTTC<br>R: GGCTGAGCTATTGAAGTATCTCACATAGGCTG
ΔN65	F: ATAGCTAGCACCATGAATCCTAAACTCATTAATGCCCTT<br>R: GGCTGAGCTATTGAAGTATCTCACATAGGCTG
ΔN68	F: ATAGCTAGCACCATGCTCATTAATGCCCTTCGGCG<br>R: GGCTGAGCTATTGAAGTATCTCACATAGGCTG
ΔN76	F: ATAGCTAGCACCATGTTTTTCTGGAGATTTATGTTCTATGG<br>R: GGCTGAGCTATTGAAGTATCTCACATAGGCTG
381X	F: CGCAAATGGGCGGTAGGCGTG<br>R: CCGTCGACTTAATATTCTTGCTTTTGTAAGAAATCCTGTATTTTG
382X	F: CGCAAATGGGCGGTAGGCGTG<br>R: CCGTCGACTTACTTATATTCTTGCTTTTGTAAGAAATCCTGTA
383X	F: CGCAAATGGGCGGTAGGCGTG<br>R: CCGTCGACTTATGTCTTATATTCTTGCTTTTGTAAGAAATCCTG
384X	F: CGCAAATGGGCGGTAGGCGTG<br>R: CCGTCGACTTACAATGTCTTATATTCTTGCTTTTGTAAGAAATCC
385X	F: CGCAAATGGGCGGTAGGCGTG<br>R: CCGTCGACTTATTATTCCAATGTCTTATATTCTTGCTTTTGTAAGAAA
395X	F: CGCAAATGGGCGGTAGGCGTG<br>R: CCGTCGACTTACATCACTACTTCTGTAGTCGTTAAGTTATATTC
N1184X	F: CGCAAATGGGCGGTAGGCGTG<br>R: GGCGTCGACCTACTTGTATGGTTTGGTTGACTTGG
G1185X	F: CGCAAATGGGCGGTAGGCGTG<br>R: GGCGTCGACCTAATTCTTGTATGGTTTGGTTGACTTG
Q1186X	F: CGCAAATGGGCGGTAGGCGTG<br>R: GGCGTCGACCTAGCCATTCTTGTATGGTTTGGTTG
L1187X	F: CGCAAATGGGCGGTAGGCGTG<br>R: GGCGTCGACCTATTGGCCATTCTTGTATGGTTTGG
S1188X	F: CGCAAATGGGCGGTAGGCGTG<br>R: GGCGTCGACCTAGAGTTGGCCATTCTTGTATGG
K1189X	F: CGCAAATGGGCGGTAGGCGTG<br>R: GGCGTCGACCTACGAGAGTTGGCCATTCTTG
V1190X	F: CGCAAATGGGCGGTAGGCGTG<br>R: GGCGTCGACCTATTTCGAGAGTTGGCCATTCTTG
M1191X	F: CGCAAATGGGCGGTAGGCGTG<br>R: GGCGTCGACCTAAACTTTCGAGAGTTGGCC
I1192X	F: CGCAAATGGGCGGTAGGCGTG<br>R: GGCGTCGACCTACATAACTTTCGAGAGTTGGCC
I1193X	F: CGCAAATGGGCGGTAGGCGTG<br>R: GGCGTCGACCTAAATCATAACTTTCGAGAGTTGGC
E1194X	F: CGCAAATGGGCGGTAGGCGTG<br>R: GGCGTCGACCTAAATAATCATAACTTTCGAGAGTTGG

N1195X	F: CGCAAATGGGCGGTAGGCGTG<br>R: GGCGTCGACCTACTCAATAATCATAACTTTCGAGAGTTG
S1196X	F: CGCAAATGGGCGGTAGGCGTG<br>R: GGCGTCGACCTAATTCTCAATAATCATAACTTTCGAGAG
F1437X	F: CGCAAATGGGCGGTAGGCGTG<br>R: GGCGTCGACCTAGAGGCTCCTCTCGTTCAGCAG
R1438X	F: CGCAAATGGGCGGTAGGCGTG<br>R: GGCGTCGACCTAGAAGAGGCTCCTCTCGTTCAG
Q1439X	F: CGCAAATGGGCGGTAGGCGTG<br>R: GGCGTCGACCTACCGGAAGAGGCTCCTCTCGTTC
A1440X	F: CGCAAATGGGCGGTAGGCGTG<br>R: GGCGTCGACCTATTGCCGGAAGAGGCTCCTCTC
I1441X	F: CGCAAATGGGCGGTAGGCGTG<br>R: GGCGTCGACCTAGGCTTGCCGGAAGAGGCTCC
S1442X	F: CGCAAATGGGCGGTAGGCGTG<br>R: GGCGTCGACCTAGATGGCTTGCCGGAAGAGGC
AmpR	F: AGCATTTATCAGGGTTATTGTCTCATGAGC<br>R: TAACCCTGATAAATGCTTCAATAATATTGAAAAAGGAAGAGT
L957K	F: AAATGTTACATTCTGTTAAGCAAGCACCTATGTCAAC<br>R:GTTGACATAGGTGCTTGCTTAACAGAATGTAACATTT
Q958K	F: GTTACATTCTGTTCTTAAAGCACCTATGTCAAC<br>R: GTTGACATAGGTGCTTTAAGAACAGAATGTAAC
P960K	F: CTGTTCTTCAAGCAAAGATGTCAACCCTCAAC<br>R: GTTGAGGGTTGACATCTTTGCTTGAAGAACAG
M961K	F: CTTCAAGCACCTAAGTCAACCCTCAAC<br>R: GTTGAGGGTTGACTTAGGTGCTTGAAG
S962K	F: CAAGCACCTATGAAAACCCTCAACACG<br>R: CGTGTTGAGGGTTTTCATAGGTGCTTG
T963K	F: GCACCTATGTCAAAGCTCAACACGTTGAAAG<br>R: CTTTCAACGTGTTGAGCTTTGACATAGGTGC
L964K	F: GCACCTATGTCAACCAAGAACACGTTGAAAGC<br>R: GCTTTCAACGTGTTCTTGGTTGACATAGGTGC
N965K	F: GTCAACCCTCAAGACGTTGAAAGCAG<br>R: CTGCTTTCAACGTCTTGAGGGTTGAC
T966K	F: CAACCCTCAACAAGTTGAAAGCAGG<br>R: CCTGCTTTCAACTTGTTGAGGGTTG
L967K	F: CCCTCAACACGAAGAAAGCAGGTGGG<br>R: CCCACCTGCTTTCTTCGTGTTGAGGG
A969K	F: CCTCAACACGTTGAAAAAAGGTGGGATTC<br>R: GAATCCCACCTTTTTTCAACGTGTTGAGG
G970K	F: CAACACGTTGAAAGCAAAGGGGATTCTTAATAGATTC<br>R:GAATCTATTAAGAATCCCCTTTGCTTTCAACGTGTTG
S1058K	F: CACTCATCTTGTTACAAAGTTAAAAGGACTATGG<br>R: CCATAGTCCTTTTAACTTTGTAACAAGATGAGTG
L1059K	F: ACAAGCAAAAAAGGACTATGGACACTTCGTG<br>R: GTCCTTTTTTGCTTGTAACAAGATGAGTGAAAATTGG
K1060R	F: AGAGGACTATGGACACTTCGTGCCTTCG<br>R: GTGTCCATAGTCCTCTTAAGCTTGTAACAAGATG
G1061K	F: GTTACAAGCTTAAAAAAGCTATGGACACTTCG<br>R: CGAAGTGTCCATAGCTTTTTTAAGCTTGTAAC
L1062K	F: CAAGCTTAAAAGGAAAATGGACACTTCGTG

	R: CACGAAGTGTCCATTTTCCTTTTAAGCTTG
W1063K	F: GCTTAAAAGGACTAAAGACACTTCGTG<br>R: CACGAAGTGTCTTTAGTCCTTTTAAGC
T1064K	F: AAGGACTATGGAAACTTCGTGCCTTC<br>R: GAAGGCACGAAGTTTCCATAGTCCTT
L1065K	F: GGACTATGGACAAAGCGTGCCTTCGGACG<br>R: CGTCCGAAGGCACGCTTTGTCCATAGTCC
N901K	F: AAAAGCTATGCAGTGATTATCACCAGCACC<br>R: CACTGCATAGCTTTTATTTCTACTATGAGTACTATTCC
S902K	F: ACAAATATGCAGTGATTATCACCAGCACCAG<br>R: TCACTGCATATTTGTTATTTCTACTATGAGTACTATTCC
Y903K	F: CAGCAAAGCAGTGATTATCACCAGCACC<br>R: GATAATCACTGCTTTGCTGTTATTTCTACTATGAGTAC
A904K	F: ACAGCTATAAAGTGATTATCACCAGCACCAG<br>R: TCACTTTATAGCTGTTATTTCTACTATGAGTACTATTCC
V905K	F: CAGCTATGCAAAGATTATCACCAGCACCAG<br>R: ATCTTTGCATAGCTGTTATTTCTACTATGAGTACTATTC
T908K	F: GCAGTGATTATCAAAAGCACCAGTTCGTATTATG<br>R: GTGCTTTTGATAATCACTGCATAGCTGTTATTTCTAC
S909K	F: GCAGTGATTATCACCAAAACCAGTTCGTATTATG<br>R: GGTGATAATCACTGCATAGCTGTTATTTCTACTATG
T910K	F: CAGTGATTATCACCAGCAAAAGTTCGTATTATGTG<br>R: GCTGGTGATAATCACTGCATAGCTGTTATTTC
S911K	F: CCAGCACCAAATCGTATTATGTGTTTTACATTTACG<br>R: AATACGATTTGGTGCTGGTGATAATCACTGC
S912K	F: CCAGCACCAGTAAGTATTATGTGTTTTACATTTACG<br>R: AATACTTACTGGTGCTGGTGATAATCACTGC
D565A	F: GAGCAGTATACAAAGCTGCTGATTTGTA<br>R: CAATCAGCAGCTTTGTATACTGCTC
D567A	F: GCAGTATACAAAGATGCTGCTTTGTATTTATTAGAC<br>R: GTCTAATAAATACAAAGCAGCATCTTTGTATACTGC
D565A/D567A	F: GCAGTATACAAAGCTGCTGCTTTGTATTTATTAGACTCTCC<br>R: GCAGTATACAAAGCTGCTGCTTTGTATTTATTAGACTCTCC
D565G	F: GCAAGAGCAGTATACAAAGGTGCTGATTTGTATTTATTAG<br>R: CTAATAAATACAAATCAGCACCTTTGTATACTGCTCTTGC

Table S8. Conditions for immunoprecipitation

<table>
<tr><th>Antibody</th><th>Mab/Pab</th><th>Species</th><th>Protein A/G</th><th>Incubation 4°C</th><th>Wash Buffer</th></tr>
<tr><td>MM13-4</td><td>Mab</td><td>Mouse</td><td>G</td><td>overnight</td><td>PBS, 0.2% SDS, 1% Triton X-100</td></tr>
<tr><td>E1-22</td><td rowspan="2">Pab</td><td rowspan="2">Rabbit</td><td rowspan="2">A</td><td rowspan="2">3 h</td><td rowspan="3">10 mM Tris-HCl pH 8.6, 300 mM NaCl, 0.05% SDS, 0.05% Triton X-100</td></tr>
<tr><td>TMD1C</td></tr>
<tr><td>3G11</td><td rowspan="2">Mab</td><td>Rat</td><td rowspan="2">G</td><td rowspan="7">overnight</td></tr>
<tr><td>7D12</td><td>Mouse</td><td>50 mM Tris-HCl pH 8.0, 150 mM NaCl, 1 mM EDTA</td></tr>
<tr><td>MrPink</td><td rowspan="2">Pab</td><td rowspan="2">Rabbit</td><td rowspan="7">A</td><td rowspan="2">10 mM Tris-HCl pH 8.6, 300 mM NaCl, 0.1% SDS, 0.05% Triton X-100</td></tr>
<tr><td>G449</td></tr>
<tr><td>570</td><td rowspan="2">Mab</td><td rowspan="2">Mouse</td><td rowspan="4">50 mM Tris-HCl pH 8.0, 150 mM NaCl, 1 mM EDTA</td></tr>
<tr><td>217</td></tr>
<tr><td>I4N</td><td rowspan="2">Pab</td><td rowspan="2">Rabbit</td></tr>
<tr><td>TMD2C</td><td rowspan="4">3 h</td></tr>
<tr><td>596</td><td rowspan="3">Mab</td><td rowspan="3">Mouse</td><td rowspan="3">20 mM MES, 100 mM NaCl, 30 mM Tris-HCl pH 7.4) with 0.5% Triton X-100</td></tr>
<tr><td>2-39.14</td><td rowspan="2">G</td></tr>
<tr><td>2-3.5</td></tr>
</table>

SUPPLEMENTARY REFERENCES

[1] Fontana A, Polverino de Laureto P, De Filippis V, Scaramella E, Zambonin M. Probing the partly folded states of proteins by limited proteolysis. Fold Des. 1997;2:R17-26.

[2] Hubbard SJ. The structural aspects of limited proteolysis of native proteins. Biochim Biophys Acta. 1998;1382:191-206.

[3] Ebeling W, Hennrich N, Klockow M, Metz H, Orth HD, Lang H. Proteinase K from Tritirachium album limber. Eur J Biochem. 1974;47:91-7.

[4] Morihara Y, Tsuzuki H. Specificity of proteinase K from Tritirachium album limber for synthetic peptides. Agr Biol Chem. 1975;39:1489-92.

[5] Kraus E, Femfert U. Proteinase K from the mold Tritirachium album Limber. Specificity and mode of action. Hoppe Seylers Z Physiol Chem. 1976;357:937-47.

[6] Kraus E, Kiltz HH, Femfert UF. The specificity of proteinase K against oxidized insulin B chain. Hoppe Seylers Z Physiol Chem. 1976;357:233-7.

[7] Whittaker RG, Manthey MK, Le Brocque DS, Hayes PJ. A microtiter plate assay for the characterization of serine proteases by their esterase activity. Anal Biochem. 1994;220:238-43.

[8] Bromme D, Peters K, Fink S, Fittkau S. Enzyme-substrate interactions in the hydrolysis of peptide substrates by thermitase, subtilisin BPN', and proteinase K. Arch Biochem Biophys. 1986;244:439-46.

[9] Laemmli UK. Cleavage of structural proteins during the assembly of the head of bacteriophage T4. Nature. 1970;227:680-5.

[10] Makowski G, Ramsby M. Protein molecular weight determination by sodium dodecyl sulfate polyacrylamide gel electrophoresis. Protein Structure The Practical Approach Series. 1997;1:1-27.

[11] Rath A, Glibowicka M, Nadeau VG, Chen G, Deber CM. Detergent binding explains anomalous SDS-PAGE migration of membrane proteins. Proc Natl Aca Sci U S A. 2009;106:1760-5.

[12] Hoelen H, Kleizen B, Schmidt A, Richardson J, Charitou P, Thomas PJ, et al. The primary folding defect and rescue of ΔF508 CFTR emerge during translation of the mutant domain. PloS One. 2010;5:e15458.

[13] Cleveland DW, Fischer SG, Kirschner MW, Laemmli UK. Peptide mapping by limited proteolysis in sodium dodecyl sulfate and analysis by gel electrophoresis. J Biol Chem. 1977;252:1102-6.

[14] Cui L, Aleksandrov L, Chang XB, Hou YX, He L, Hegedus T, et al. Domain interdependence in the biosynthetic assembly of CFTR. J Mol Biol. 2007;365:981-94.

[15] Peters KW, Okiyoneda T, Balch WE, Braakman I, Brodsky JL, Guggino WB, et al. CFTR Folding Consortium: methods available for studies of CFTR folding and correction. Methods Mol Biol. 2011;742:335-53.

[16] Mendes F, Farinha CM, Roxo-Rosa M, Fanen P, Edelman A, Dormer R, et al. Antibodies for CFTR studies. J Cyst Fibros. 2004;3 Suppl 2:69-72.

[17] Kleizen B, van Willigen M, Mijnders M, Peters F, Grudniewska M, Hillenaar T, et al. Co-translational folding of the first transmembrane domain of ABC-transporter CFTR is supported by assembly with the first cytosolic domain. J Mol Biol. 2021:166955.

[18] Drozdetskiy A, Cole C, Procter J, Barton GJ. JPred4: a protein secondary structure prediction server. Nucleic Acids Res. 2015;43:W389-94.

[19] Liu F, Zhang Z, Csanady L, Gadsby DC, Chen J. Molecular structure of the human CFTR ion channel. Cell. 2017;169:85-95 e8.

[20] Kim S, Skach W. Mechanisms of CFTR folding at the endoplasmic reticulum. Front Pharmacol 3: 201. 2012.
